# Light-evoked activity and BDNF regulate mitochondrial dynamics and mitochondrial localized translation in CNS axons

**DOI:** 10.1016/j.isci.2025.113563

**Published:** 2025-09-15

**Authors:** Alexander Kreymerman, Jessica E. Weinstein, Nirmal Vadgama, Sahil H. Shah, Michael M. Nahmou, Kinsley C. Belle, Marco H. Ji, Xin Xia, Anne Faust, Yolandi Van Der Merwe, David N. Buickians, In-Jae Cho, Star K. Huynh, Sonya Verma, Kristina Russano, Xiao-Lu Jin, Ioannis Karakikes, Michael B. Steketee, Jeffrey L. Goldberg

**Affiliations:** 1 Spencer Center for Vision Research, Byers Eye Institute, Stanford University, Palo Alto, CA 94303, USA; 2 University of Miami Miller School of Medicine, Miami, FL 33136, USA; 3 Cardiovascular Institute, Stanford School of Medicine, Stanford, CA 94303, USA; 4 Department of Cardiothoracic Surgery, Stanford School of Medicine, Stanford, CA 94305; 5 Medical Scientist Training Program, University of California, San Diego, San Diego, CA 92093, USA; 6 Department of Ophthalmology and McGowan Institute for Regenerative Medicine, University of Pittsburgh, Pittsburgh, PA 15213, USA

**Keywords:** Natural sciences, Biological sciences, Neuroscience, Systems neuroscience

## Abstract

Mitochondria coordinate well-described maintenance functions within neuronal axons and dendrites. However, less is known about how mitochondria are regulated during axon development and maturation. Here, we demonstrate that within the developing visual system, retinal ganglion cell (RGC) axons in the retina and optic nerve exhibit increases in mitochondria size, number, and total area *in vivo*. Our findings indicate that these developmental changes in mitochondria are driven by neuronal activity associated with eye opening and by brain-derived neurotrophic factor (BDNF). These events occur in concert with downstream gene and protein expression changes consistent with mitochondrial biogenesis and energetics pathways. We further demonstrate that activity- and BDNF-regulated transcripts are localized and translated at mitochondria within RGC axons *in vivo*, concomitant with the regulation of mitochondrial dynamics. These data highlight the previously undescribed regulation of mitochondrial dynamics in axonal maturation, dependent on mechanisms involving neuronal activity and neurotrophic factor signaling, coordinated with mitochondrial-localized translation.

## Highlights

• Mito. dynamics in RGC axons are regulated by eye opening, visual evoked activity, and BDNF
• Activity and BDNF regulate the expression of nuclear encoded RNAs and mito. dynamics genes
• Activity and BDNF regulated mito. RNA binding and local translation events in axons
• Mito.-bound nuclear RNAs encode genes involved in axon maturation and growth pathways

## Graphical abstract

**Figure:**
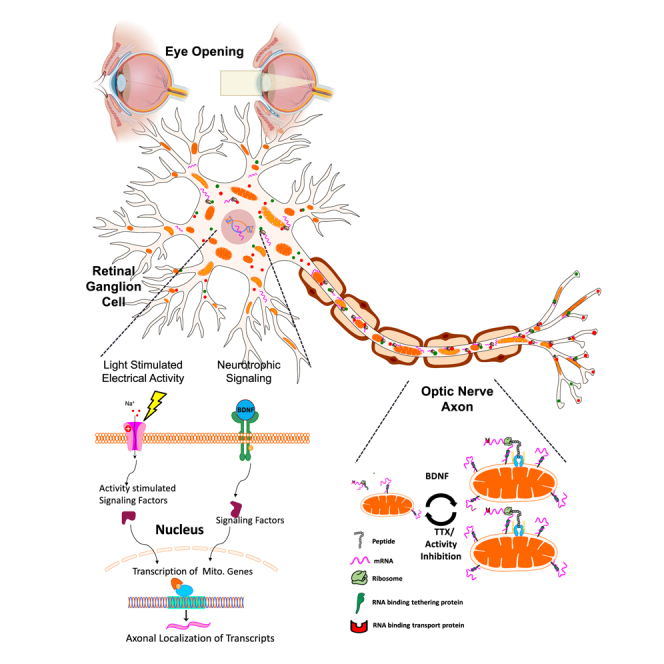

## Introduction

Neurons are among the most metabolically active cell types in the body, and mitochondria, which generate ATP through oxidative phosphorylation can be located far from the cell soma.^1^^,^^2^^,^^3^^,^^4^ Mitochondria also support neurons and their extensive axonal compartments through activities such as calcium homeostasis, fatty acid oxidation, and modulation of secondary messengers and signaling pathways.^5^ These mitochondrial functions rely on the expression and assembly of approximately 600–1500 proteins encoded in the nucleus.^6^^,^^7^^,^^8^^,^^9^^,^^10^^,^^11^^,^^12^^,^^13^ To maintain continuous communication between mitochondria and the nucleus, neurons shuttle mitochondria up and down axons using kinesin and dynein motor proteins.^14^^,^^15^^,^^16^^,^^17^ Transported mitochondria can also undergo fission or fusion with neighboring mitochondria, acquiring or shedding genetic material and proteins in the process.^18^ Additionally, new mitochondria can be assembled and packaged with nuclear and mitochondrial-encoded proteins through mitochondrial biogenesis, which occurs both in the perinuclear space and within axons, leading to increased mitochondria numbers in neuronal compartments.^19^^,^^20^^,^^21^ Together, these changes in mitochondrial localization, size, number, and total cellular complement are referred to as mitochondrial dynamics.

Supplying nuclear-encoded proteins to distal axonal mitochondria can also leverage transport and local translation of RNA in axonal compartments.^22^ Interestingly, many studies indicate that a significant portion of axon-localized transcripts encode nuclear proteins that regulate mitochondrial functions.^23^^,^^24^^,^^25^^,^^26^ Nuclear-encoded mitochondrial transcripts have been shown to physically localize on or within mitochondrial membranes,^27^^,^^28^^,^^29^^,^^30^^,^^31^ with further evidence suggesting that mitochondria can serve as local translation sites.^32^^,^^33^^,^^34^^,^^35^ However, the regulation of this localization is not well understood. Here, we demonstrate that developmental changes in axonal mitochondria are regulated by neuronal activity *in vivo*, and define the associated regulation of mRNA expression and localization to mitochondria by activity in retinal ganglion cells (RGCs) *in vitro* and *in vivo*.

## Results

### Mitochondrial networks reorganize concomitant with eye opening

**Figure 1:**
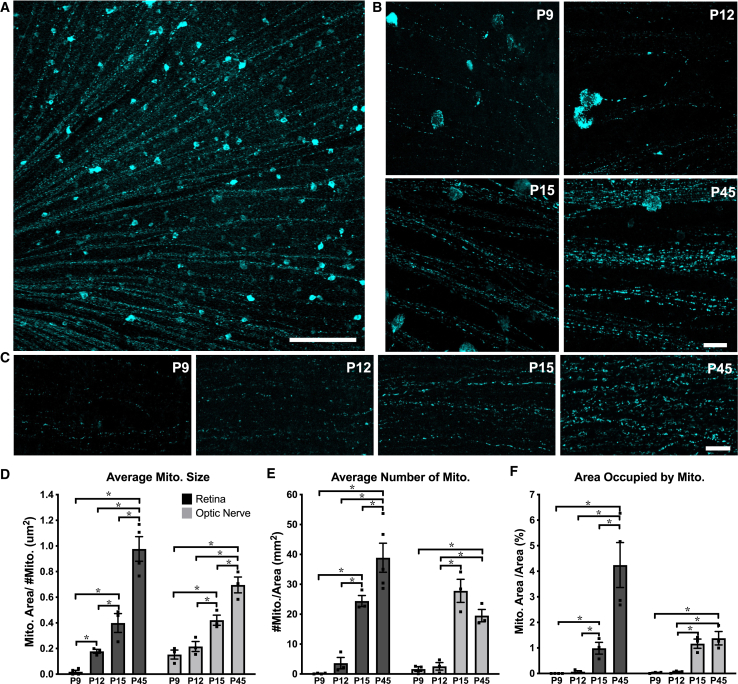
Axonal mitochondria increase in size, number, and area through eye development CFP+ mitochondria were imaged by confocal microscopy and analyzed in ImageJ. (A) Example maximum intensity projected image of a whole mounted P45 retina showing mitochondrial labeling within RGC cell bodies (circular objects) and axonal segments (linear segments) diving toward the optic nerve head (200 μm scale bar). (B) Example images of retinal, and (C) optic nerve axon mitochondria from postnatal day 9 (P9), P13, P15, and P45 mice (20 μm scale bar). (D–F) In both retinal and optic nerve RGC axons, the average mitochondrial size, number, and area (measured as percent of cross sections, representing fractional volume) increased from P9 to adulthood (Error bars indicate SEM; *N* = 4 replicate mice for P9 ages, *N* = 3 for P12 and P15, and *N* = 4 for P45 mice, 9 images analyzed per animal; one-way ANOVA with Holm-Sidak correction for multiple comparisons, ∗*p* ≤ 0.05). See Figure S1 for image analysis procedures.

We first studied mitochondrial organization in RGC axons in transgenic mice expressing cyan fluorescent protein (CFP) fused to the Cox8a mitochondrial targeting sequence under control of the *Thy1* promoter (*Thy1*-CFP/Cox8a).^36^^,^^37^^,^^38^ *Thy1* promoter-related artifacts in mitochondrial CFP signal are unlikely in our imaging data*,* as *Thy1* gene expression peaks around P12 in RGCs and continues to be stably expressed throughout adulthood.^39^ In this mouse, approximately 5% of RGCs express the CFP/Cox8a transgene, permitting the visualization and quantification of mitochondria in RGC axons (Figure 1A–1C). We used this mouse model to investigate axon-specific mitochondrial networks at postnatal (P) days 9, 12, 15, and 45, as RGCs experience significant developmental changes through this time period.^28^^,^^29^^,^^30^^,^^31^^,^^40^^,^^41^^,^^42^^,^^43^^,^^44^^,^^45^^,^^46^^,^^47^^,^^48^ Of note, these time points also follow the period of developmental cell death in RGCs, which peaks at P5 in mice,^49^^,^^50^^,^^51^ thus allowing for the identification of mitochondrial changes independent of cell death signaling, which can influence mitochondrial morphology.^52^^,^^53^ Analysis of CFP-labeled mitochondria in whole mount retinas and optic nerves by confocal microscopy revealed significant reorganization in RGC axons throughout postnatal development (Figures 1B and 1C). Overall, mitochondria increased in size, number, and occupied a greater percentage of axonal area from P9 to P45 (Figures 1D–1F). Interestingly, within a relatively short window of development, around eye opening (P12/13 to P15), mitochondrial size, number, and occupied area increased in both RGC retinal and optic nerve axon segments, with optic nerve mitochondria experiencing the greatest change during this time window.

**Figure 2:**
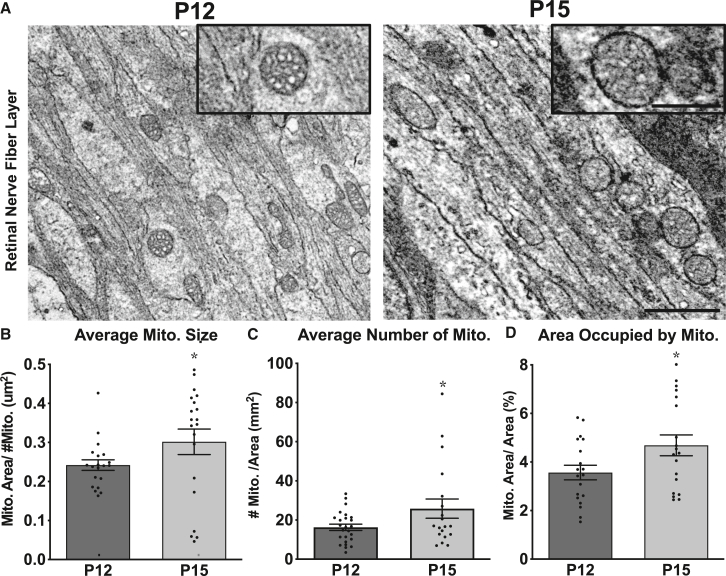
Mitochondrial size and number increase at eye opening in RGC axons (A) Mitochondria were imaged and quantified in retinal nerve fiber layer RGC axons by transmission electron microscopy before (P12) and after (P15) eye opening. Increased magnification (insets, 500 nm scale bar) shows mitochondrial membrane enfolding and cristae, and representative mitochondrial size differences (full panel,1 μm scale bar). (B) Mitochondrial size, (C) number, and (D) area increased significantly between P12 and P15. (Error bars indicate SEM; *n* = 20 replicate sections per age, ≥150 traced mitochondria per age; significance identified by Student’s t test, ∗*p* < 0.05).

Because fluorescence data per tissue area in Figure 1 does not specifically consider axon area in the calculation, we set out to confirm our findings using transmission electron microscopy and imaging mitochondria at time points in which metrics changed the most, P12 and P15 (Figure 2). TEM has the advantage of minimizing potential artifacts associated with the resolution limits of light microscopy, for example, the identification of individual mitochondria that at the light level could be counted as networked larger mitochondria. At these time points, TEM measurements recapitulated trends found by confocal imaging, with absolute mitochondrial values differing slightly from confocal measures, presumably introduced by greater resolution capabilities of EM and from differences in tissue processing.^54^ Nonetheless, data replicates mitochondrial size, number, and occupied area increase by 25%, 59%, and 31% respectively, from P12 to P15 in RGC axons (Figures 2B–2D).

### Eye opening regulates mitochondrial networks

**Figure 3:**
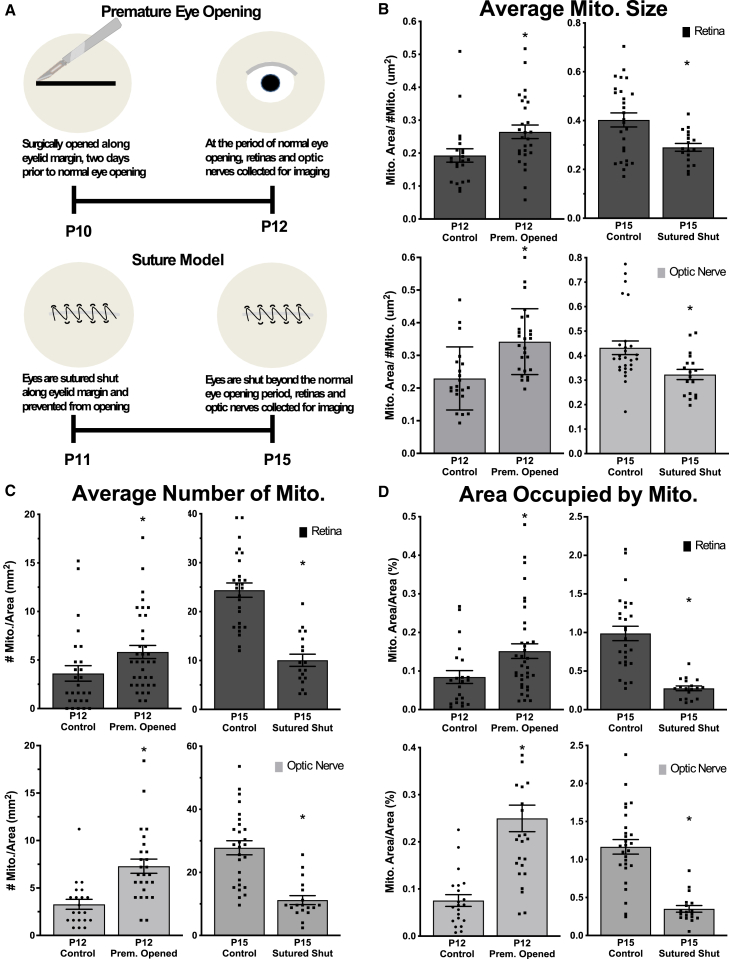
Eye opening is sufficient and necessary for developmental changes in mitochondrial size and localization (A) Surgical model for premature eye opening and sutured eyelid closure. (B) Average mitochondrial size, (C) number, and (D) area increase with premature eye opening, and this developmental increase is inhibited by prolonged eye closure. (Points are averages from ≥18 images analyzed per condition; 9 images per replicate animal *N* = 3, except P15 sutured samples *N* = 2. Error bars indicate SEM. Student’s t test, ∗*p* < 0.05.).

As eye opening occurs between P12-P15 with concomitant increases in visual activity,^55^ we asked whether mitochondrial morphological changes are caused by or dependent on eye opening by surgically inducing premature or delayed eye opening in CFP-Cox8a mice (Figure 3A). For premature eye opening, we opened the P10 eyelid margin, two days prior to normal eye opening, and allowed animals to mature to P12. We found increases in mitochondrial size, number, and occupied area in retinal and optic nerve axons compared to unopened P12 eyes (Figures 3B–3D). Thus, eye opening accelerates the mitochondrial morphology changes identified in normal development.

Conversely, to determine if eye opening is necessary for the developmental increases in mitochondrial size, number, and occupied area from P12-15, we delayed eye opening by suturing eyelids shut at P11, prior to natural eye opening, then allowed mice to mature to P15, delaying eye opening by 2–3 days (Figure 3A). Compared to age-matched controls, delayed eye opening led to significant decreases in all mitochondrial measurements (Figures 3B–3D), in most cases back to levels found in P12 animals. Thus, these data suggest that the process of eye opening is sufficient and necessary for the mitochondrial network increases found from P12-15.

### Retinal ganglion cell activity and brain-derived neurotrophic factor regulate mitochondrial networks

**Figure 4:**
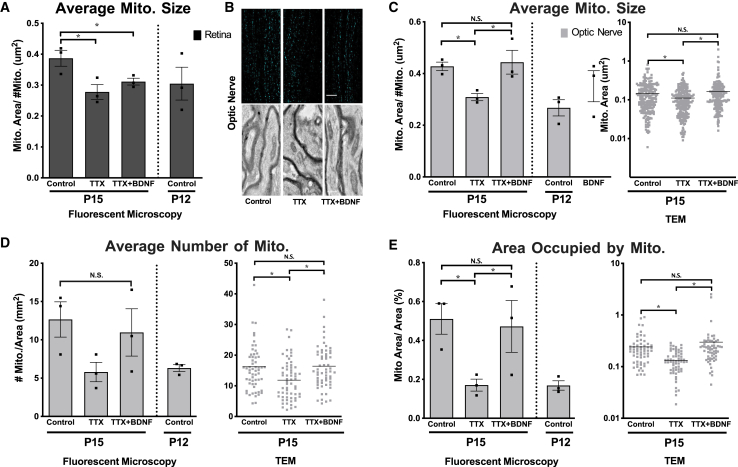
Mitochondrial developmental changes are dependent on retinal electrical activity and are partially rescued by BDNF in optic nerve axons Vehicle Control, TTX- or TTX plus BDNF-treated mice analyzed at P15, along with vehicle Control injected mice analyzed at P12. (A) Measured changes in average mitochondrial size within retinal axons by confocal microscopy. (B) Representative images of mitochondria within optic nerve axons of Control, TTX, and TTX plus BDNF-treated eyes, fluorescence confocal microscopy images in upper panels (presented as a maximum intensity projection, scale bar 5 μm) and transmission electron microscopy in lower panels (TEM, presented as crop of a 4000x full field image, scale bar 500 nm). Axons were identified by myelination and neurofilament in TEM images. Corresponding mitochondrial (C) size, (D) number, and (E) area measurements from fluorescent microscopy and TEM (Error bars indicate SEM; *N* = 3 replicate eyes per condition, 9 images analyzed per eye in fluorescent imaging measurements; *n* > 60 sections from *N* = 2 replicated eyes per condition, >200 traced mitochondria in TEM measurements; significance detected using one-way ANOVA with Holm-Sidak corrections for multiple comparisons, ∗*p* < 0.05). See Figure S2 for TEM tracings and quantification procedures.

These findings suggested the hypothesis that light-stimulated electrical activity in axons (i.e., action potentials) contributes to the observed changes in axon mitochondrial distribution and morphology. To test the contribution of electrical activity to axonal mitochondrial changes during the period of eye opening, we pharmacologically inhibited both spontaneous and light-evoked electrical activity in RGCs by intravitreally injecting tetrodotoxin (TTX)^56^ just prior to normal eye opening at P11 and again after eye opening at P13, followed by mitochondrial quantification at P15 by both fluorescent confocal microscopy and TEM. Note that TTX has previously not been found to disrupt RGC survival or axon number when injected through these age ranges.^57^ We found that TTX but not control vehicle injection inhibited mitochondrial increases in size within both retinal and optic nerve axons (Figures 4A–4C), and mitochondrial number and occupied area only in the optic nerve portion of RGC axons (Figures 4D and 4E), to similar levels found in vehicle control injected P12 optic nerves. Thus, RGC electrical activity is a significant contributor to mitochondrial network changes that occur in association with eye opening. However, discrepancies in mitochondrial numbers and area within retinal versus optic nerve axons likely indicate additional regulation contributing to mitochondrial dynamics during this period.

downstream of electrical activity, BDNF expression has been shown to be regulated by eye opening during this time period, and to be blocked by TTX injection,^40^^,^^58^^,^^59^^,^^60^^,^^61^^,^^62^^,^^63^ and in separate assays to modulate mitochondrial dynamics.^40^^,^^64^^,^^65^^,^^66^^,^^67^ To determine whether BDNF could rescue the inhibitory effects of TTX on mitochondrial networks, BDNF and TTX were co-injected at P11 and again at P13, and mitochondrial parameters were measured at P15. BDNF was capable of significantly reversing the TTX-induced decreases in mitochondrial size, number and occupied area in optic nerve axons (Figures 4C–4E). Of note, this rescue effect of BDNF was only detected in the optic nerve but not retinal axons (Figure 4A), suggesting again that different mechanisms regulate mitochondrial dynamics in a compartment-specific manner within the axon. Together, these data suggest that activity and BDNF are both critical in regulating the morphology and distribution of optic nerve axon mitochondria during this stage of visual system development.

### Activity and brain-derived neurotrophic factor regulate the expression of nuclear encoded mitochondrial genes

**Figure 5:**
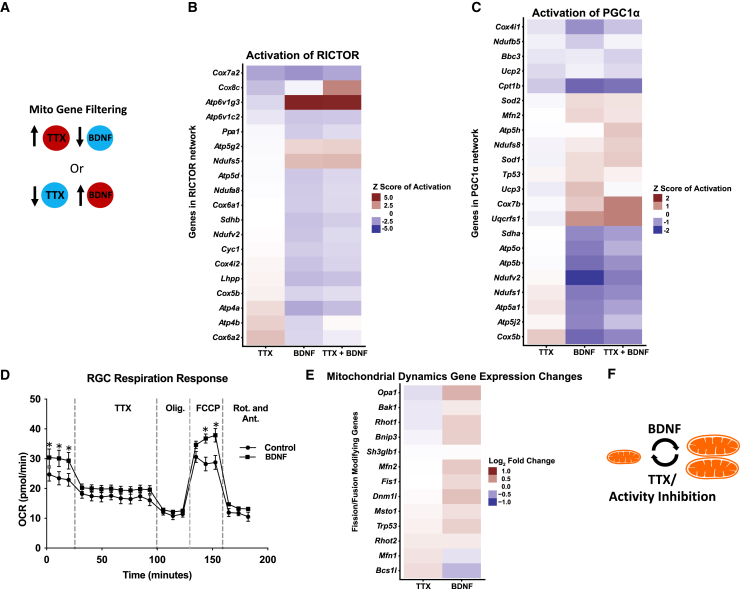
RGC nuclear-encoded mitochondrial gene expression in response to activity inhibition with TTX and/or BDNF is consistent with inhibition versus activation of mitochondrial dynamics and energetics (A) Model for filtering data acquired from RT-PCR gene array analysis of P15 acutely purified RGCs, after TTX and/or BDNF intravitreal injections at P11 and P13 (*N* = 3 RGC preps per condition). Filters were placed to identify gene expression regulated in opposing directions by BDNF and TTX. The resulting genes were then passed through IPA pathway analysis software, which suggested 10 major upstream regulators, with PGC1-α and RICTOR at the top of the list. Downstream gene expression data modulated by these upstream regulators were transformed into *Z* score of activation. Up- or downregulated gene sets are denoted by color. (B) Genes identified in our array that are regulated by RICTOR represent mainly energetics genes. (C) Genes identified in our array that are regulated by PGC1-α represent mainly mitochondrial dynamics and biogenesis regulators. (D) Measuring the effect of BDNF on mitochondrial dependent oxygen consumption in purified RGCs shows an increase in the basal respiration rate and maximum respiration capacity (with FCCP addition) regardless of activity inhibition by TTX (introduced 35min after initial recording). TTX, Oligomycin, FCCP, and Rotenone/Antimycin A, were added sequentially at times points marked with vertical lines. Recorded values were acquired using the Seahorse XF96 instrument (Error bars indicate SEM; *n* = 6 replicates per condition, pooled from 3 separate RGC preps and assayed on one plate, ∗ indicate *p* ≤ 0.05, T-test). (E) Genes identified in our array that have been previously demonstrated as mitochondrial fission/fusion or mitochondrial size modifying are opposingly regulated by TTX and BNDF, with most genes upregulated by BDNF. (F) Model of the predicted mitochondrial events triggered by TTX or BDNF, based on gene pathway analysis and the identified mitochondrial changes in injected mice.

To investigate molecular mechanisms associated with activity and BDNF’s dramatic regulation of axonal mitochondria dynamics, we explored the transcriptional influence of exogenously added BDNF and TTX on nuclear-encoded mitochondrial gene expression in RGCs. To accomplish this, we injected TTX and/or BDNF in combination or alone at P11 and P13, acutely purified RGCs from P15 retinas, and extracted RNA for qRT-PCR gene arrays. We then analyzed expression data and conducted pathway analysis. Major upstream regulators were identified by probing the gene expression sets for targets of known regulators, and then filtering for genes whose expression was concordant with the inhibitory effects of TTX and subsequent rescue with BDNF on mitochondrial morphology and distribution (Figure 5A). The resulting analysis revealed that PGC1-⍺ and RICTOR, master modulators of mitochondrial dynamics and energetics, were putative upstream regulators of genes modulated by TTX and/or BDNF (Figures 5B and 5C). For many mitochondrial genes regulated by RICTOR and PGC1-⍺, TTX and BDNF showed opposing effects on expression. In most cases, the gene expression profile of TTX+BDNF mimicked that of BDNF alone, suggesting BDNF’s downstream signaling events are dominant and capable of overriding TTX effects. Furthermore, BDNF modestly increased basal and maximum respiratory capacity in purified RGCs *in vitro,* even in the presence of TTX (Figure 5D), consistent with pathways predicted by these gene expression changes. Ontological analysis of these gene expression datasets suggested opposing functions between BDNF and TTX, with fission/fusion and mitochondrial biogenesis pathways induced by BDNF and suppressed by TTX (Figures 5E and 5F), consistent with our *in vivo* data.

### Activity regulates mitochondrial-associated local translation and mitochondrial dynamics

**Figure 6:**
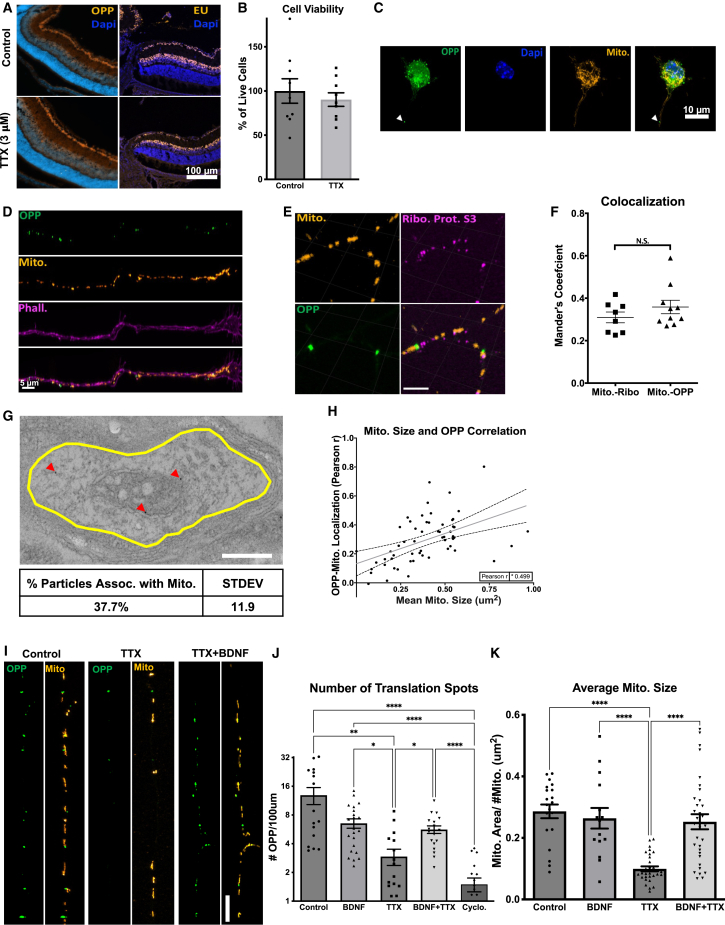
Activity regulates mitochondrial localized protein translation in axons (A) Representative images collected of P15 retinas, after *in vivo* intravitreal injections of TTX or BSS (control) at P11 and P13, and an injection of EU or OPP 2 h before tissue processing. (B) Cell viability of TTX treated RGCs, identified as Calcein-AM positive and Sytox negative, and normalized to total cell number by Hoescht. Graphed as a percent change relative to control cells (Error bars indicate SEM; 9 replicates; Student’s t test). (C) Confocal images of cultured RGCs, virally labeled with mitochondrial targeted dsRed and pulsed OPP for 15 min before fixation. OPP is detected in RGC cell bodies and in axons. (D) Axon tips demonstrate OPP puncta throughout, growth cones, and terminal axon domains. Axon tips labeled with phalloidin in far red. (E) Representative image of mitochondria, OPP, and ribosomal protein S3 colocalization within axons (scale bar 5 μm). (F) Mander’s coefficient values for colocalizations (error bars indicate SEM; *n* = 9 blind selected axons from *N* = 3 replicate RGC preps; one-way ANOVA with Holm-Sidak’s t-test). (G) TEM evidence of ribosomal protein S3 localization with mitochondria in axons, *in vivo*. The yellow circle represents an axonal area identified by ensheathing myelin structures, and the red arrows show all detectable S3 puncta identified by gold antibody labeling. (H) Correlation of OPP-mitochondrial colocalization relative to mitochondrial size (regression line and 95% confidence intervals are plotted, data points from *n* = 30 blind selected cells selected across *N* = 3 replicate RGC preps). (I) Representative images of mitochondrial and OPP-labeled axons in vehicle control, TTX, and TTX+BDNF treated RGCs (scale bar 5um). (J) Number of OPP puncta per 100 μm of axon termini, treated with BDNF, TTX, TTX+BDNF, cycloheximide, and vehicle controls (error bars indicate SEM; >10 blind selected axons from replicate RGC preps; one-way ANOVA with Tukey’s multiple comparisons test, ∗*p* < 0.05). (K) Mean mitochondrial size in RGC axons incubated with TTX, BDNF, TTX+BDNF, or vehicle controls (error bars indicate SEM; >10 blind selected axons from replicate RGC preps; one-way ANOVA with Dunn’s multiple comparisons test, ∗*p* < 0.05). See Figures S3–S6, for example, EU labeling and thresholding or masks used in mitochondrial area, OPP spot counts, colocalization, and TEM analyses.

Our gene arrays showed that the expression of many of the mitochondrial genes assayed was suppressed by the TTX-mediated inhibition of activity. To investigate whether transcription or translation activity was being globally downregulated in RGCs by activity inhibition, we treated RGCs with 5-ethynyluridine (EU), a uridine analog, or O-propargyl-puromycin (OPP), a puromycin analog. These molecules readily incorporate into newly synthesized RNAs (with EU) or proteins (with OPP), and can be conjugated to fluorophores with click chemistry to visualize the location and relative amount of synthesis taking place within cells.^68^^,^^69^ Using this approach, we first tested whether TTX inhibited transcription or translation by intravitreally injecting TTX at P11 and P13, and then pulsing with EU or OPP at P15 for 1h. Upon visualization, no detectable differences in signal intensity from EU or OPP were identified in retinas (Figure 6A), suggesting that transcription and translation where not broadly inhibited by activity suppression.

We followed up *in vivo* experiments with *in vitro* approaches where EU and OPP labeling can be better controlled and quantified. RGCs were isolated to 99% purity by immunopanning from early postnatal mouse retinas and seeded at low density,^70^ allowing for the visualization of individual cells and neurites. Then, cells were virally transduced to fluorescently label mitochondria with a Cox8a-targeted dsRed, cultured for 48 h, treated with TTX (1 μM) for 2 h, and finally pulsed for 15 min with OPP or 1 h with EU. The cells were treated with TTX for a shorter period of time than during *in vivo* experiments to avoid significant changes in viability, which at early postnatal days has the potential to decrease cell survival, unlike the *in vivo* time points tested.^57^^,^^71^ Without performing extensive *in vivo* versus *in vitro* pharmacodynamic TTX half-life experiments, no detectable decrease in viability after 2 h of TTX incubation was detected (Figure 6B) during a window in which TTX is active, as previously demonstrated.^71^^,^^72^ In purified primary RGCs, we again observed cells with intensely labeled peri-nuclear regions, but no discernable differences in new transcript or protein levels in the cell body regions (Figures 6C and S3), similar to the data derived in RGCs *in vivo*. Interestingly, in OPP-treated RGCs, obvious puncta were also visible in axonal segments. These puncta appeared scattered throughout distal axon segments in axonal tips, with varying sizes and numbers, and in close proximity to mitochondria (Figure 6D).

Axon-localized protein translation has been previously described,^30^ but it is unknown whether activity modulation can regulate these events or whether local translation can occur on mitochondria in mammalian cells. We examined whether RGC axon-identified OPP puncta are indeed representative of local translation activity, if these events occur on mitochondria, and whether activity modulation via TTX and/or BDNF is capable of modifying local translation. Immunostaining of cytoplasmic ribosomal protein S3, an integral component of the 40s-ribosomal subunit translation initiation site,^73^ showed colocalization with OPP (Figure 6E), and ribosomal protein S3 colocalized with mitochondria to a similar degree as OPP puncta with mitochondria (Figures 6E and 6F), providing strong evidence that the detected OPP puncta are likely local translation sites at mitochondria. By TEM, we confirmed that ribosomes bind mitochondria in RGC axons *in vivo*, and that approximately 37% (±11.9%) of axonal-localized ribosomes are associated with outer mitochondrial membranes, as measured in P15 optic nerve samples (Figures 6G and S6). Interestingly, we also found that increases or decreases in mitochondrial size significantly correlated with increases or decreases in OPP-to-mitochondria localization (0.499 r, Figure 6H).

Given the TTX and BDNF treatments’ regulation of mitochondrial size, we asked whether these treatments would also regulate axon-localized OPP puncta. Quantifying the abundance of OPP sites within axonal segments, we found that TTX-treated axons demonstrated significant (>70%) decreases in the number of OPP puncta as compared to controls, similar to the effects of translational inhibitor cycloheximide, and again BDNF partially rescued these TTX-induced decreases (Figures 6I and 6J). Changes in OPP puncta again correlated with mitochondrial size alterations, with TTX reducing mitochondrial size and BDNF mitigating these events (Figure 6K), similar to *in vivo* data. Of note, changes in these captured translation events are not likely due to decreased transport of newly synthesized proteins from the cell body, as the rate of soluble protein transport is less than 0.1 μm/s (or 90 μm in 15 min) and would be an unlikely captured change within the OPP assay window (15 min incubation) in distal RGC axons,^74^ which are typically longer than 600 μm after 48 h of culture in these cells.^75^ Thus, taken together, these data support a model in which activity and BDNF regulate local protein translation in RGC axons, at or near mitochondria, associated with the regulation of the mitochondrial network during development.

### RNA binding proteins tether nuclear-encoded mitochondrial mRNA to mitochondria

**Figure 7:**
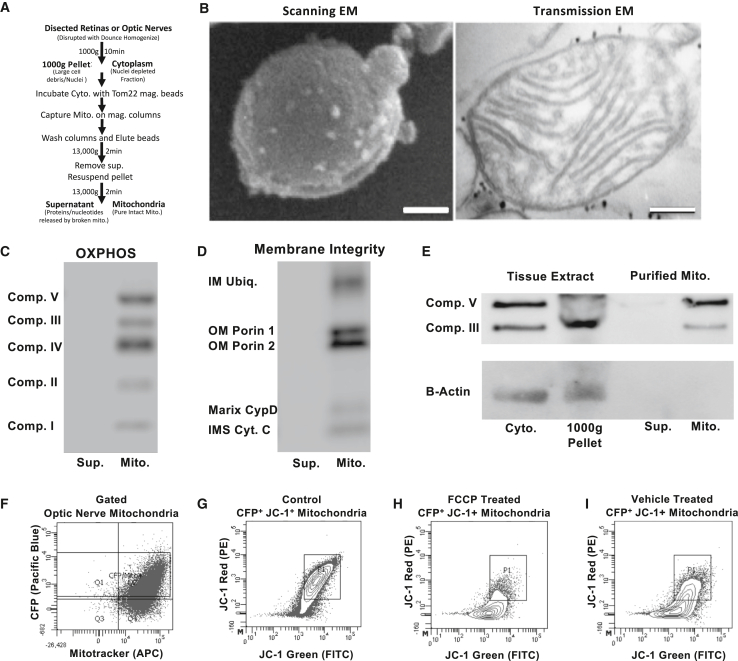
Purified mitochondria retain their protein content and membrane integrity (A) Outline of mitochondrial isolation and subsequent assays. (B) TOM22-bound nanoparticles are visible bound to the outer mitochondrial membrane in both SEM (white spots) and TEM (black dots). Scale bars 50 nm and 100 nm. (C–E) Western blot analyses of purified mitochondria and supernatants. Magnetically isolated mitochondria retain (C) OXPHOS subunits, as well as (D) outer membrane (OM), inner membrane (IM), and inner membrane space (IMS) proteins. (E) B-Actin is detectable in cytoplasmic, 1000g fractions, but not mitochondrial isolate fractions. (F) FACS assayed mitochondria confirm the intact and functional state of mitochondria, which retain CFP and membrane potential-dependent mitotracker CMXROS. (G) Sorted CFP+ mitochondria also demonstrate polarization-dependent fluorescence with JC-1, and (H) lose membrane potential with FCCP depolarization to a greater degree than (I) vehicle-treated controls. See Figure S7 for uncut versions of blots and the cytoplasmic GAPDH blot.

To investigate whether the identified OPP and ribosomal protein S3 mitochondrial localization have the potential to act as docking sites for nuclear-encoded mRNA and new protein translation, we conducted qRT-PCR and proteomic experiments on mitochondria purified from optic nerves or retinas. To accomplish this, we dissected, homogenized, and incubated whole optic nerve or retina tissue lysates with a magnetically conjugated Translocase Of Outer Mitochondrial Membrane 22 (TOM22) antibody, to allow the magnetic selection of mitochondria (Figure 7A).^76^ Isolated mitochondria were first examined by SEM and TEM, which showed that mitochondrial membranes and cristae architecture were structurally maintained after isolation, with dark puncta visible on the outside of mitochondria (Figure 7B), representing nanoparticle-bound TOM22, and confirming the integrity of outer mitochondrial membranes after isolation. By Western blot, isolated mitochondria maintained proteins from all complexes in the electron transport chain (ETC; Figure 7C) and inner and outer membrane integrity proteins (Figure 7D), but no significant detectable levels of cytoplasmic B-actin or GAPDH (Figures 7E and S7C). Of note, supernatant fractions from TOM22-purified mitochondria, which would reflect TOM22-bound membranes from ruptured, non-intact mitochondria, had no detectable ETC or membrane integrity proteins (Figures 7C–7E), suggesting that nearly all TOM22-purified mitochondria were intact and captured in the pellet fraction.

To further purify RGC-specific mitochondria from these tissue-derived mitochondria fraction, TOM22-selected optic nerve mitochondria isolated from *Thy1*-CFP/Cox8a mice by fluorescence-activated cell sorting (FACS). In FACS assays, mitochondria from *Thy1*-CFP/Cox8a mice retained membrane integrity and CFP positivity, detected by mitotracker-CMXROS co-staining (Figure 7F). Furthermore, isolated CFP^+^ mitochondria maintained high membrane potentials and readily took up the mitochondrial polarization integrity dye JC-1, forming distinct populations of red-shifted J-aggregate-retaining mitochondria (Figure 7G) that lost polarization in response to a membrane potential uncoupler, carbonyl cyanide-*4*-(trifluoromethoxy) phenylhydrazone (FCCP), as compared to vehicle treated fractions (Figures 7H and 7I). Overall, these data demonstrate that optic nerve mitochondria isolated via magnetic and FACS selection deliver pure and structurally intact mitochondria, with surface and internal proteins and functional polarization maintained throughout the procedure.

**Figure 8:**
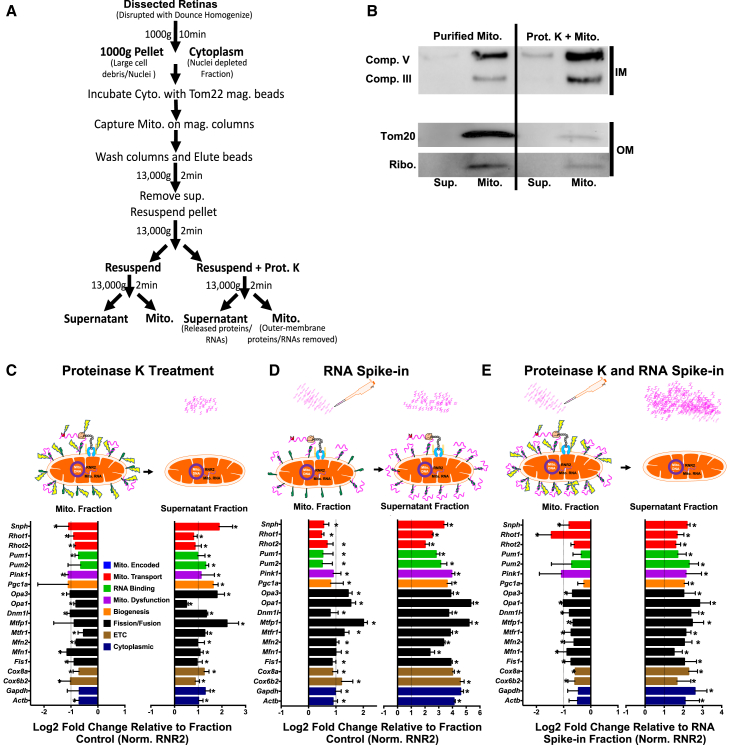
Purified mitochondria bind nuclear-encoded mRNAs through outer membrane-associated proteins (A) Outline of mitochondrial isolation protocol and subsequent assays. For downstream blots and qPCR experiments, just prior to assigning group (i.e., control and proteinase K treated mitochondria), purified mitochondria were resuspended and split into equal volumes for equal starting material in subsequent work flow. (B) Relative to control mitochondrial pellets, proteinase K treated fractions retain inner membrane Comp. III and Comp. V proteins, but have reduced outer membrane (OM) TOM20 proteins and associated ribosomes (Ribo., Ribosomal Protein S3). (C) Proteinase K-treated mitochondria release bound RNA into supernatant fractions, as detected by qPCR of pelleted mitochondrial fractions and corresponding supernatant fractions. (D) Total retinal RNA extracts spiked into purified mitochondrial fractions lead to increased mitochondria-RNA binding and RNA in supernatant fractions. (E) Mitochondrial RNA binding decreases despite RNA spike-in, when proteinase K is also applied. Illustrated outcomes presented above all datasets. Data is normalized to RNR2 expression and graphed as a log2 fold change relative to stated fractional controls (*N* = 3 replicate retina mitochondrial purifications, dotted vertical line is equivalent to a 2-fold change, statistical significance for individual gene fold changes were identified by Student’s t test against control fraction/baseline values, p∗<0.05). See Figure S7 for whole blots.

We then used these mitochondrial isolates to determine whether nuclear-encoded mRNA is found on mitochondria. To accomplish this we purified mitochondria from P15 mouse retinas, extracted total RNA from mitochondrial pellets, and assayed for the presence of nuclear-encoded mRNA by RT-qPCR arrays, after treating mitochondrial isolates with or without proteinase K to release surface proteins (Figure 8A). Of note, this procedure is a well-established approach for probing surface proteins on mitochondria,^77^^,^^78^^,^^79^^,^^80^^,^^81^ and allows for an unbiased approach to distinguish RNA binding via proteins rather than by random or non-specific stickiness. Validating this approach, we found that proteinase K treatment selectively digested proteins off outer mitochondrial membranes, as shown by western blot with the reduced presence of outer membrane protein TOM20, and the retention of inner membrane ETC proteins, complex III protein (ubiquinol-cytochrome c reductase) and complex V protein (ATP synthase alpha-subunit) (Figure 8B). As suggested by our immunofluorescence and EM data, we also found ribosomal protein s3 detectable on purified mitochondrial fractions by western blot but reduced after proteinase K treatment, thus providing additional support for ribosomal tethering and RNA binding on outer mitochondrial membranes.

We then assayed for a range of nuclear-encoded mitochondrial regulators (as in Figure 5), including genes that regulate mitochondrial dynamics, cell death, and energetics. All detected mRNAs amplified in less than 30 cycles, lending confidence to the integrity of target detection (Figures S8A and S8B). Samples treated with proteinase K demonstrated significant mRNA release from mitochondria into supernatant fractions, relative to controls (Figure 8C). To further test the dependence on proteins for RNA-mitochondrial tethering, we supplemented purified mitochondrial preps with 200 ng of total RNA purified from whole retinas (“spike-in” controls). In mitochondrial fractions spiked with RNA we detected a modest but significant 2-fold increase in nuclear-encoded mitochondrial mRNAs and, as expected, a significantly larger 8- to 16-fold increase in supernatant fractions relative to controls (Figure 8D). However, when mitochondria were treated with proteinase K in combination with spike-in RNA, we detected an approximately 2-fold decrease in the amount of mRNA bound to mitochondrial fractions and a 2– to 4-fold increase in mRNA released into the supernatant, relative to RNA spike-in fraction controls (Figure 8E). Thus, even with the exogenous addition of RNA to purified mitochondrial fractions, there is a clear dependence on RNA binding proteins, digestible by proteinase K, for RNA-mitochondria interactions. The released mRNAs coded for proteins known to regulate mitochondrial dynamics, biogenesis, energetics, and RNA transport. However, we also found mRNA encoding cytoplasmic proteins GAPDH and actin bound to mitochondria, suggesting that bound mRNAs are not limited to those coding for mitochondrial-specific proteins.

**Figure 9:**
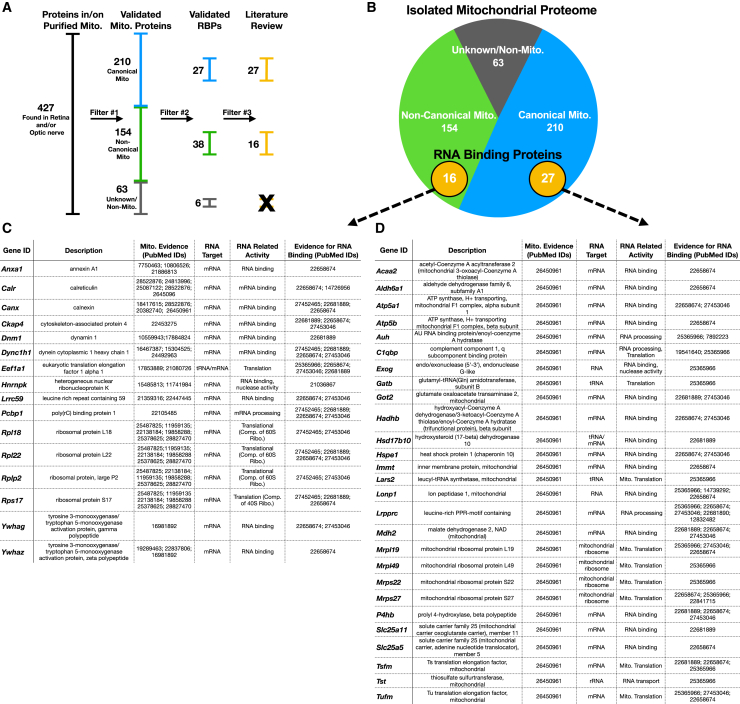
Proteomics mass spectrometry analysis reveals nuclear-encoded RNA binding proteins associated with purified mitochondria (A) Filtering used to identify mitochondria-specific proteins. (B) Venn diagram of total protein hits sorted by annotation in the MitoCarta database. (C and D) Candidate mitochondria-associated RNA binding proteins with cited evidence (PubMed ID shown) for their functional RNA binding role and mitochondrial interaction (displayed data is pooled from *N* = 3 replicate retina mitochondrial purifications and *N* = 3 replicate optic nerve mitochondrial purifications; all displayed proteins were considered significant/present as they were detected in one or more replicate preps and with one or more peptides mapped with >95% confidence.

We then examined what proteins are candidates for RNA binding to mitochondria by performing mass spectrometry on replicate mitochondrial purifications from optic nerve and whole retinas. This yielded a total of 427 identifiable proteins after analyzing and pooling the results from 6 replicate preps (3 retinal and 3 optic nerve mitochondrial isolations). We cross-referenced these identified proteins with MitoCarta2.0, a non-visual-system-derived database of proteins that co-purify with mitochondria,^9^^,^^11^ and stratified proteins into three groups based on evidence of mitochondrial localization (Figures 9A and 9B): 210 canonical mitochondrial proteins based on MitoCarta data from proteomics, computation, and microscopy analyses; 154 non-canonical mitochondrial proteins in the MitoCarta database that correlate with less pure mitochondrial fractions; and 63 proteins that do not show up in the MitoCarta database and thus may be non-mitochondrial, or may be visual system-specific mitochondrial proteins.

Among these externally validated mitochondria-associated proteins, we identified the subset with predicted RNA binding by David,^82^ Panther,^83^ and Uniport^84^ databases using the gene ontology term “RNA binding.” Proteins that met these criteria were then cross-referenced against datasets from the RNA-protein interactome of cardiac cells,^85^ HEK cells,^86^ and HeLa cells,^87^ as well as RNA binding protein databases AtTRACT and RBPDB.^88^^,^^89^^,^^90^^,^^91^ This yielded the identification of 71 mitochondrial-associated RNA-binding proteins, of which 43 had published evidence of direct interaction with mitochondria (Figures 9C and 9D). These included nuclear-encoded proteins with known intra-mitochondrial roles, which may also act as RNA binding proteins inside or outside the mitochondria, as well as translation, RNA processing, and RNA shuttling proteins. Overall, these data lay a foundation for future hypothesis testing and highlight an underlying interaction in which mitochondria bind nuclear-encoded mRNAs, known to modulate mitochondrial size, number, and energetics, and that this process is mediated by RNA binding proteins present on mitochondrial membranes.

### mRNAs regulating mitochondrial dynamics are attached to mitochondria and altered by activity and brain-derived neurotrophic factor in retinal ganglion cell axons

**Figure 10:**
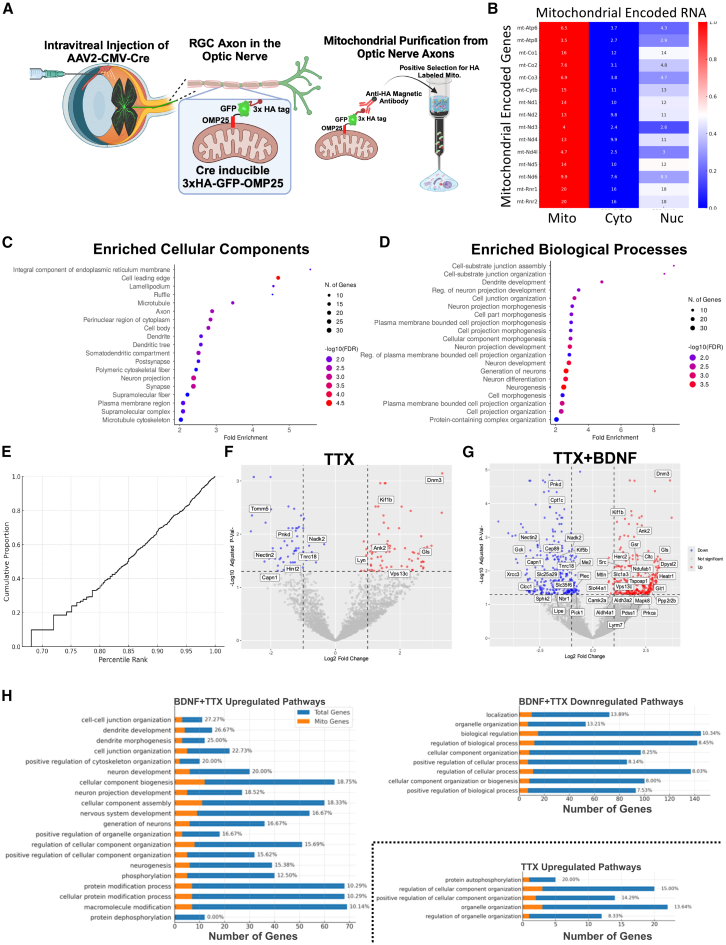
RNA-seq of purified axonal mitochondria reveals co-purifying nuclear-encoded RNAs with roles in axonal growth, maintenance, and mitochondrial regulation that are activity modulated (A) Schematic workflow of sample preparation. Intravitreal injection of AAV2 virus expressing Cre into the Mito-Tag mice drives the expression of a non-functional Cre-inducible 3xHA tagged mitochondrial outer membrane protein 25 (OMP25). Using the anatomical separation of the intravitreal retinal space and RGC axons passing through the optic nerve, mitochondria from axons are purified by pulling down HA-tagged mitochondria from dissected, homogenized, and HA magnetic antibody-incubated optic nerve lysates. (B) Heatmap shows the distribution of mitochondrial-encoded genes in isolated mitochondria and cytoplasm or nuclear fractions, demonstrating mitochondrial enrichment in the purifications used for analysis. The heatmap includes normalized expression values, with higher values indicating greater enrichment. (C) Dot plot of enriched cellular components for the top 200 nuclear encoded genes in the BSS-HA sample, indicating the -log10(FDR) significance and number of genes involved. Each dot represents a specific cellular component, with the size of the dot corresponding to the number of genes and the color intensity indicating the significance level. (D) Dot plot of enriched biological processes for the top 200 nuclear encoded genes in the BSS sample, showing -log10(FDR) significance and the number of genes involved. Each dot represents a specific biological process, with the size of the dot corresponding to the number of genes and the color intensity indicating the significance level. (E) Cumulative proportion of mitochondrial-related genes found on axonal mitochondria plotted against the percentile rank of overall gene expression. The plot shows the distribution of expression ranks, with higher percentiles indicating higher expression levels. (F) Volcano plot comparing TTX vs. Controls, highlighting mitochondrial-related genes. The plot shows log2 fold change on the x axis and -log10 *p*-value on the y axis, with significant genes (*p* < 0.05 and logFC >1 or < −1) highlighted. (G) Volcano plot comparing BDNF+TTX vs. Controls, with mitochondrial-related genes labeled. The plot shows log2 fold change on the x axis and -log10 *p*-value on the y axis, with significant genes (*p* < 0.05 and logFC >1 or < −1) highlighted. (H) Three bar graphs illustrate the pathways involved in different conditions and the corresponding number of genes implicated. Blue bars represent the total number of genes involved in each pathway, while orange bars indicate the number of mitochondrial genes. The percentage of mitochondrial genes is displayed at the end of each bar, with pathways ordered by the increasing proportion of mitochondrial genes.

To further investigate the regulation of transcripts tethered to mitochondria (as in Figure 8), we performed RNA-seq on RGC optic nerve axon mitochondria after TTX and BDNF. Taking advantage of updated technological approaches to purify cell-specific mitochondria, we switched to the Mito-Tag mouse, which expresses a CRE inducible, GFP- and HA-linked nonfunctional outer mitochondrial membrane protein tag, allowing for cell-specific mitochondrial isolation using anti-HA antibody-conjugated magnetic beads, as previously demonstrated.^92^ To activate the mitochondrial tag in RGCs, we used the intravitreal injection of an AAV2-expressing CRE. Two weeks post-injection, we dissected optic nerves and isolated mitochondria from RGC axonal projections with anti-HA magnetic beads (Figure 10A), as in the above, validated, anti-TOM22-based isolations. RNA-seq showed mitochondrial DNA-encoded RNAs (Figure 10B), demonstrating mitochondrial capture, and nuclear-encoded mRNAs, confirming prior data.

Ranking the abundance of nuclear-encoded RNAs found on purified mitochondria, the top 200 enriched transcripts encoded genes that were associated with neuronal projections and compartments, such as lamellipodia, cytoskeletal components, plasma membranes, and synapses (Figure 10C). Notably, traditional mitochondrial or bioenergetic categories were not dominant in the GO enrichment analysis of the top 200 transcripts (Figure 10D), due in large part due to the exclusion of mtDNA-encoded genes in the analysis. However, the top-ranked transcripts found on axonal mitochondria collectively participate in axonal structure, growth, morphogenesis, and synapse-related functions (Figures 10C and 10D), indicative of the broader functional repertoire of axonal mitochondria and consistent with a role for mitochondria as local translation platforms for axonal functions. To confirm that nuclear encoded mitochondrial-related transcripts were nevertheless enriched on axonal mitochondria, we plotted the cumulative expression percentile distribution of nuclear mitochondrial-related transcripts relative to all transcripts detected (Figure 10E), which showed a clear upward shift relative to a random distribution, indicating the overrepresentation of mitochondrial transcripts among the high-abundance transcripts found in our preps. A Mann-Whitney U test comparing the percentile ranks of mitochondrial and non-mitochondrial genes further validated this distribution (*p* = 2.42 × 10^−24^). Thus, there is some specificity of mitochondrial-associated mRNAs toward mitochondrial- and axon-associated proteins.

To determine if activity modulation via TTX and/or BDNF administration affects these nuclear-encoded mitochondrial-associated mRNAs, we administered intravitreal doses of TTX, BDNF, or TTX with BDNF as in Figure 4, two weeks after CRE injection. We then purified HA-tagged RGC axonal mitochondria from optic nerves for RNA extraction and sequencing. In these samples, TTX significantly changed the levels of a subset of transcripts in mitochondria (69 increased and 54 decreased), which included genes for proteins that are both mitochondrial-regulating and non-mitochondrial-regulating, compared to saline-injected controls (Figure 10F). BDNF alone had no effect on mitochondrial transcript localization, but, similar to our *in vivo* and *in vitro* data, in the presence of TTX co-administration, significantly changed the levels of a greater number of transcripts on mitochondria (291 increased and 260 decreased), also showing greater amplitude of change (Figure 10G). These results demonstrate that transcript localization to mitochondria is regulated by activity and BDNF.

Pathway analysis revealed that TTX+BDNF co-treatment upregulated transcripts on axonal mitochondria in pathways related to neuronal projection regulation and organelle organization (Figure 10H). Some pathways were regulated in opposite directions: TTX-upregulated but TTX+BDNF downregulated pathways relevant to “mitochondrial organization,” which correlates with the observed developmental changes in mitochondrial dynamics identified above. These data demonstrate that activity modulation regulates the association of nuclear-encoded transcripts with axonal mitochondria. Of particular interest are the potential roles of mitochondrial-tethered transcripts in mitochondrial dynamics, axonal maintenance, and function, which warrant further investigation into whether these captured transcripts and their local translation are critical to intra-axonal mitochondrial function and axon maintenance.

## Discussion

Proper CNS neuron development and homeostasis depend on mitochondrial organization and function throughout long axons. Mitochondria must dynamically change to meet axonal demands far from the cell body. As a result, neurons and their axons have particularly demanding requirements for the active expression, trafficking, and assembly of nuclear-encoded mitochondrial macromolecules (proteins and mRNAs). Here, we build upon known mechanisms regulating rapid mitochondrial changes in axons and present new findings that activity and BDNF modulate developmental mitochondrial dynamics (size, number, and total area) in axons of the developing visual system, coordinated with the mitochondrial based local translation of nuclear encoded RNAs responsible for axonal growth, architecture, and synapse-related functions.

Furthermore, we demonstrate that eye opening, activity, and BDNF modulation of mitochondrial dynamics, along with local translation events occur *in vivo* and *in vitro* with specificity to mitochondria in RGC axons, using a variety of approaches. We provide evidence that *in vivo* mitochondrial dynamics changes occur throughout development and around eye opening, correlated with activity and BDNF responses, by utilizing the Thy1-CFP/Cox8a mice. These mice have been shown by us and others to provide consistent fluorescent labeling of mitochondria within RGC axon in the nerve fiber layer of retinas and throughout the optic nerve.^36^^,^^37^^,^^38^ We validated these fluorescent level mitochondrial changes against high resolution electron microscopy measures, utilizing well established regional and ultrastructure landmarks, such as myelination, axoplasm, microtubules, and neurofilament,^93^^,^^94^^,^^95^^,^^96^^,^^97^^,^^98^ to ensure RGC axonal specific mitochondrial changes.

We also provide data that *in vivo* eye opening, activity, and BDNF driven mitochondrial dynamics alterations are correlating with RGC specific transcriptional and metabolic changes. *In vitro* we show that neural activity and BDNF in RGC axons act as inputs to modulate a novel mechanism of mitochondrial localized translation in coordination with mitochondrial dynamics that mimic *in vivo* data. We ensure that in these assays the captured transcripts are purified from RGCs and that local translation events are imaged in RGC axons, using a previously established immunopanning procedure that provides highly purified RGCs from *in vivo* samples.^70^^,^^99^^,^^100^ We then expand on *in vitro* findings by demonstrating that mitochondria can act as local translation sites, providing evidence of nuclear encoded RNA and RNA binding protein tethering to mitochondria, from *in vivo* purified mitochondria. Of note, while mitochondrial tethered RNAs and RNA binding proteins identified in purified mitochondria are from whole retinas or optic nerve samples, thus ensuring sample quantity to satisfy assay conditions, we also go on to validate nuclear encoded transcript tethering with specificity to RGC mitochondria.

Using the mito-tag mouse,^92^ compartmentalized intravitreal injections of AAV2 expressing CRE virus,^101^^,^^102^ and distal dissection of optic nerve tissue, we were able to selectively purify optic nerve RGC axon mitochondria using HA tags. Using this approach, in combination with the intravitreal injection of TTX and BDNF, allowed us to selectively capture RGC axon specific effects of activity and BDNF on nuclear encoded transcripts tethering to mitochondria, *in vivo.* Ultimately, we reveal that this novel activity and the BDNF-modulated mechanism of nuclear transcript tethering in axonal mitochondria carry potential regulatory roles in axon development and structural maturation. Furthermore, our data support that mitochondrial nuclear mRNA binding and localized translation activity are robust in the visual system and are likely applicable to other CNS cell types. We discuss these findings in greater detail and delve into the implications of our investigation for understanding mitochondrial and local translation mechanisms in neurodevelopment, axonal regeneration, and neurodegeneration in CNS diseases.

### Activity and brain-derived neurotrophic factor regulate axonal mitochondria during development

With eye opening, the visual system experiences a dramatic increase in RGC electrical activity and BDNF expression, triggered by Ca^+2^ signaling and the activation of CREB-mediated transcription.^64^^,^^103^^,^^104^^,^^105^ Both activity and BDNF play pivotal roles in the visual system at these ages^40^^,^^58^^,^^59^^,^^60^^,^^61^^,^^62^^,^^63^ and in axon development more generally, including axon growth and presynaptic maturation, with mitochondrial dynamics and energetics having stereotyped roles in these developmental events.^106^ Yet, the link between activity or BDNF signaling and changes in mitochondrial dynamics during CNS axon maturation *in vivo* had not been investigated. Here, we find that RGC activity and downstream BDNF signaling during eye opening are sufficient and necessary to increase mitochondrial size and number in RGCs’ optic nerve axons during development. Activity also plays a similar, albeit more muted, role in regulating mitochondrial morphology in RGCs’ retinal axon segments. This could be indicative of unique mitochondrial regulatory mechanisms in retinal versus optic nerve axon segments, such as local BDNF expression, TrkB receptor localization or signaling, effects on or of oligodendrocytes, or myelination.^64^^,^^107^ Knocking out BDNF or TrkB could begin to address these questions, but such experiments would be confounded by indirect effects on mitochondria downstream of BDNF’s well-described regulation of cellular and synaptic physiology,^108^ RGC survival, and axon growth.^109^ Future experiments might explore whether this increase in axonal mitochondrial size, number, and area plays a significant role only in the requisite increase in energy/ATP generation concomitant with the increase in activity and action potentials, or also in other elements of axon function such as calcium homeostasis, synaptic activity, or other physiology in the retina versus the optic nerve, relating to development or degeneration.^110^^,^^111^

### Activity regulates mitochondrial dynamics-associated gene expression

We also implicate a mechanism in which activity regulates nuclear-encoded transcript expression related to mitochondrial biogenesis, energetics, and dynamics. Analysis of mitochondrial regulatory gene expression pointed to activity- and BDNF-mediated activation of transcriptional networks linked to PGC1-⍺ and RICTOR during eye opening, with TTX and BDNF administration (whether in the presence of TTX or not) producing opposing gene expression patterns. In addition, we find that BDNF mediates increases in basal and maximum respiratory capacity of RGCs, even if TTX is present, also reflecting the potential functional influences of known cellular energetics links with increased PGC1-⍺ and RICTOR signaling.^112^^,^^113^^,^^114^ These data, taken together with the in vivo regulation of mitochondria by activity and BDNF, suggest that corresponding activity- and BDNF-modulated PGC1-⍺ and RICTOR transcriptional pathways are likely critical contributors to RGC axonal mitochondrial activities during eye opening and RGC development. The functional relevance of specific genes identified within PGC1-⍺, RICTOR, and other mitochondrial genes will need to be explored in future studies. As much is left to be learned about the interplay between mitochondrial fission and fusion genes, for example, in regulating mitochondrial dynamics, it is hard to predict how the identified combinations of specific gene changes should increase or decrease the dynamics regulated here, necessitating empirical studies in future work.

### Activity regulates a novel mechanism of mitochondrial localized translation

Our investigation also implicates a novel mechanism in which activity- and BDNF-regulated mitochondrial dynamics act in concert with intra-axonal nuclear-encoded transcript tethering and translation at mitochondria. Nuclear proteins are translated in the perinuclear space and transported down axons at rates of up to 0.3 mm/h for soluble proteins, 4 mm/h when associated with mitochondria, or 16 mm/h with neuropeptide-containing vesicles,^74^^,^^115^^,^^116^ which may not be quick enough to resupply proteins at distal axonal sites in times of rapid demand. Alternatively, nuclear-encoded mRNAs, including some for mitochondrial proteins^23^^,^^24^^,^^25^ are also known to be transported and pooled in distal axon sites within RGC axons.^26^ These mRNAs can then be locally translated under normal conditions or in response to local demand.^22^

**Figure 11:**
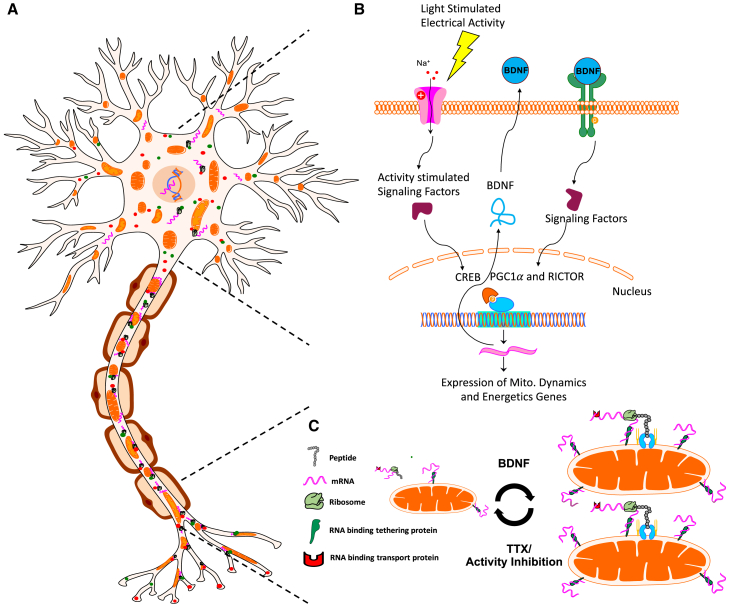
A model for activity- and BDNF-regulated mitochondrial size, number, and associated protein translation (A) Neurons contain mitochondria, RNA, RNA binding and transport proteins, ribosomes, and newly synthesized proteins, throughout distal axon and dendrite compartments. (B) Electrical activity in RGCs, for example, driven by the light stimulation of retinal circuitry after eye opening, activates a signaling pathway that culminates in the activation of transcription factors such as CREB, and the expression of BDNF. BDNF signaling stimulates nuclear-encoded mitochondrial gene expression, coordinated by the activation of transcriptional regulators RICTOR and PGC1-α. (C) Neuronal activity and downstream BDNF signaling stimulate increases in mitochondrial size and number, reversed by activity inhibition. Changes in mitochondrial size triggered by activity modulation and BDNF also correlate with modifications in nuclear-encoded transcripts localization and mitochondrial localized translation events.

Consistent with our findings, activity modulation and neurotrophic factors, including BDNF, have been shown to regulate local translation in Xenopus neurons.^117^^,^^118^ Our study extends these findings by showing that such translation occurs at mitochondria in an activity-dependent manner, here studying RGCs *in vitro* and *in vivo*. Our data reveal ribosomes bound to RGC axon mitochondria by light and electron microscopy, demonstrate the enrichment of nuclear mRNAs encoding mitochondrial and axonal proteins on mitochondria in axons by RNAseq, identify RNA binding proteins on RGC mitochondria by proteomics, and quantify activity and BDNF’s co-modulation of axonal mitochondrial size, number, and area (*in vitro* and *in vivo*) and local translation at or near mitochondria by OPP labeling. Together, these data suggest a novel mechanism not previously described whereby increased neuronal firing and BDNF downstream signaling pathways regulate mitochondrial dynamics in part through modified local translation (Figure 11).

These data also point to the importance of the axonal translation of both nuclear-encoded mitochondrial and even non-mitochondrial RNAs, as not all mitochondrial-bound RNAs encode proteins specific to mitochondria. For example, despite evidence for non-detectable or very low levels of actin protein in mitochondrial purifications by us and others,^119^^,^^120^^,^^121^ the translation of actin RNA in axons has been shown to associate with mitochondria and coordinate axon branching.^122^ Similarly, GAPDH RNA has also been found bound to mitochondria,^123^ although protein levels are low or not significantly detectable at mitochondria under normal conditions, unless cells are undergoing apoptosis.^124^ In our RNA-seq data, we also find other transcripts not previously identified in mitochondrial compartments, i.e., GTPase, motor, kinase, cell adhesion, enzyme, and ubiquitin-regulating transcripts. In addition, our findings that axonal mitochondria associate with mRNAs involved in axon growth and morphogenesis and not solely with canonical mitochondrial transcripts reflect the multifunctional roles mitochondria may play in developing axons. This supports the emerging model in which mitochondria serve as scaffolds for the localized translation of nuclear-encoded transcripts necessary for axonal growth, structural organization, and possibly even synaptic assembly. Thus, it will be important to identify the contribution of mitochondrial localization for specific transcripts as it relates to their axonal function, and also the relative contribution of transcription-versus local translation-mediated regulation. Nonetheless, our data support a model in which neuronal activity and BDNF both signal to modify the mitochondrial localization of nuclear transcripts, local translation events, and mitochondrial dynamics, with BDNF able to rescue or alter these activity-dependent features.

### Limitations of this study and implications for future studies

Our data ultimately provide a road map for investigating whether neuronal activity-modulated mitochondrial dynamics and mitochondrial-based local translation play a role in other periods of development (e.g., in target branching or synapse formation), aging, after injury, or in neurodegeneration.^110^ Furthermore, while we focus our work on identifying evoked activity roles in modulating mitochondrial dynamics, spontaneous activity, particularly during early visual system axonal growth, is also likely modulating mitochondrial dynamics, as suggested by mitochondrial changes from P9-12. Indeed, it is known that RGC viability and apoptotic axonal refinement events occur with a dependence on spontaneous activity in visual system targets.^71^^,^^125^^,^^126^^,^^127^^,^^128^^,^^129^^,^^130^ Thus, future work investigating mitochondrial dynamics regulated by retinal and target site spontaneous activity events, could provide for developmentally critical axonal apoptotic signaling mechanisms through the modulation of mitochondrial dynamics.

Beyond development, defective mitochondrial transport, biogenesis, and function have also been linked to many neurodegenerative diseases.^131^ In RGC specific degenerative diseases, axon transport and particularly mitochondrial transport have been implicated in both axonal degeneration, neuroprotection, and axon regeneration.^38^^,^^132^^,^^133^^,^^134^ Given this, it will be also interesting to test whether the mitochondrial tethering of translation machinery and/or RNA binding proteins is critical to axonal survival and even regeneration. For example, does mTOR activation, a previously identified mitochondrial associating protein and axonal translation regulator, critically depend on mitochondrial tethering to exert its potent axon regeneration activity^114^^,^^135^^,^^136^^,^^137^? Future experiments delocalizing mRNAs away from mitochondria, with tools such as competitive peptides or RNA sequences designed to disrupt the RNA binding proteins found in our proteomics data, or knockdown of RNAs found in our RNA-seq data, may shed light on specific nuclear-encoded RNA or proteins critical to mitochondrial-scaffolded translation and how they contribute to axon growth or regeneration. Extending the results of our work toward approaches to better understand axon mitochondria-specific mRNAs and translation changes may ultimately yield new treatment approaches, whether in CNS axon dysfunction during development, aging, disease, injury, and even for axon regeneration therapies.

## Resource availability

### Lead contact

Requests for further information and resources should be directed to and will be fulfilled by the lead contact, Jeffrey L. Goldberg (jlgoldbe@stanford.edu)

### Materials availability

This study did not generate new unique reagents.

### Data and code availability

• All data reported in this article will be shared by the lead contact upon request.
• This article does not report original code.
• Any additional information required to reanalyze the data reported in the article is available from the lead contact upon request.

## Acknowledgments

We gratefully acknowledge funding from the NIH R01-EY020913 (JLG), P30-EY014801 (University of Miami), P30-EY026877 (Stanford University), F31-NS087789 (AK), as well as unrestricted grants from Research to Prevent Blindness, Inc. We thank the following for their technical support; John J. Perrino and Ruth Yamawaki (NIH 1S10RR02678001), as well as Peggy Bates for electron microscopy, George McNamara and Gabe Gaidosh for microscopy, Eleut Hernandez for animal husbandry, Oliver Umland for assistance in flow cytometry, and Majid Ghassemian for mass spectrometry support.

## Author contributions

Conceptualization and rational, A.K., J.W, and J.G.; writing, review, and editing, A.K., J.W., N.V., M.S., and J.G.; data curation, analysis, and graphical representation, A.K., J.W., N.V., S.H., M.N., K.B., M.J., and M.S. Surgeries and injections for *in vivo* fluorescent mitochondria imaging and quantification, J.W., *in vivo* TEM, EDU, and OPP imaging and analysis, A.K., A.F., Y.M., and M.S.; *In vitro* imaging, data analysis, and curation, A.K., D.B., I.C., S.H., and FACs data analysis and curation, A.K. and M.S.; mitochondrial isolation for proteomics, RNA binding, RNA seq., and analysis and advising, A.K., N.V., and I.K. *In vivo* injections and tissue dissection for mito.-tag experiments, A.K., M.N., and X.X; rt-qPCR data capture and analysis, S.H. and X.J.; Animal husbandry and genotyping, S.V. and K.R.; funding acquisition, J.G.

## Declaration of interests

The authors declare no competing interests.

## STAR★Methods

### Key resources table

**Table:** 

REAGENT or RESOURCE	SOURCE	IDENTIFIER
**Antibodies**		

Ribosomal Protein S3	Cell Signaling Technology	D50G7
MTCO1	Abcam	ab14705
ATP5A	Abcam	ab14748
NDUFB8	Abcam	ab110242
SH3B	Abcam	ab14714
UQCRC2	Abcam	ab14745
VDAC1	Abcam	ab14734
TOM20	Abcam	ab78547
Ubiquinol Cytochrome *c* Reductase Core Protein I	Abcam	ab110252
Cytochrome *c*	Abcam	ab110325
Complex Va	Abcam	ab110273
Cyclophilin 40	Abcam	ab110324
GAPDH	Cell Signaling Technology	2118S
B-Actin	Cell Signaling Technology	8457
Anti-HA MicroBeads	Miltenyi Biotec	130-091-122
Gold conjugated anti-rabbit	Abcam	ab27234
Alexa 647 conjugated anti-rabbit	Thermo Fisher Scientific	A-21245

**Bacterial and virus strains**		

CellLight™ Mitochondria-RFP, BacMam 2.0	Thermo Fisher Scientific	C10601
AAV2-CMV-Cre	Addgene	105537-AAV2

**Chemicals, peptides, and recombinant proteins**		

BDNF	Peprotech	450–02
TTX	Abcam	ab120055
Cycloheximide	EMD Millipore	239764
Poly-D-lysine	Sigma Aldrich	P-6407
Laminin	Sigma Aldrich	L-6274
Vecta-Shield	Vector Labs	H-1400
Mitotracker CMXROS	Thermo Fisher Scientific	M7512
JC-1	Thermo Fisher Scientific	T3168
Alexa Fluor® 647 Phalloidin	Thermo Fisher Scientific	A22287
FCCP	Sigma Aldrich	C2920
Protease inhibitors	Thermo Fisher Scientific	78425
Proteinase K	Thermo Fisher Scientific	25530049
Neurobasal media	Thermo Fisher Scientific	21103–049
Seahorse Assay Media	Agilent	102365–100
BSS	Alcon	0065079550

**Critical commercial assays**		

Click-iT Nascent RNA Assay Kit	Thermo Fisher Scientific	C10327
Click-iT Nascent Protein Synthesis Assay Kit	Thermo Fisher Scientific	C10456
Mitochondrial stress test kit	Agilent	103015–100
Mitochondria Isolation Kit	Miltenyi Biotec	130-096-946
RNeasy Plus Micro Kit	Qiagen	74034
RT^2^ Profiler™ PCR Arrays	Qiagen	PAMM-087ZE and PAMM-008ZE
iScript™ cDNA Synthesis Kit	BioRad	1708891
TaqMan Gene Expression Master Mix	Thermo Fisher Scientific	4369016

**Experimental models: Organisms/strains**		

B6.Cg-Tg(*Thy1*-CFP/Cox8a)S2Lich/J mice	The Jackson Laboratory	007967
B6.Cg-Gt(ROSA)26Sor^tm1(CAG−EGFP)Brsy/^J mice	The Jackson Laboratory	032290
C57BL/6 mice	Charles River Laboratories	C57BL/6NCrl

**Software and algorithms**		

Zen software	Zeiss	
Imaris Software	Bitplane	
Volocity software	Perkin Elmer	
ImageJ	The NIH	
WinList software	Verity Software House	
MASCOT	Matrix Sciences	
Protein Pilot 4.0	ABSCIEX	
R software	The R Foundation	
Prism	Graphpad	

### Experimental model and study participant details

#### Animal use and breeding

Experiments conformed to the ARVO Statement for the Use of Animals in Ophthalmic and Vision Research and were approved by the Stanford University Biosafety Committee and the Institutional Animal Care and Use Committee. Strains used in these experiments included wild type/cousins or littermate control offspring from CD1 crossbred to B6.Cg-Tg(*Thy1*-CFP/Cox8a)S2Lich/J or B6.Cg-Gt(ROSA)26Sor^tm1(CAG−EGFP)Brsy/^J mice (The Jackson Laboratory), as well as CFP-expressing and MitoTag mice offspring. MitoTag mice were maintained as homozygotes for all sequencing experiments, in accordance with Jackson labs. Both females and males mice were used in experiments and not distinguished in the data analysis.

#### Primary cell culture

RGCs were purified from male and female postnatal day 1/2 (P1/2) C57BL/6 mice (Charles River Laboratories) by immunopanning, and cultured on poly-D-lysine- (10 μg/mL; Sigma Aldrich, P-6407) and laminin-coated (2 μg/mL; Sigma, L-6274) cover glass bottom 96 well plates (Greiner Bio-One),^70^^,^^132^ in media with or without BDNF supplement as previously described.^86^ As standard procedure, mouse litters consisted of mixed sexes and combined in RGC purifications, ensuring sufficient RGC purification yields, reduced animal burden, and sex independent data capture for *in vitro* experiments.

### Method details

#### Eyelid opening, injections, or suturing

For premature eyelid opening experiments or injections prior to eye opening, P10 or P11 mice were anesthetized as above and eyelids were gently pried open with forceps as described.^138^ Eyes were then injected with indicated treatment and/or treated with sterile 2.5% hydroxypropyl methylcellulose (Goniosol, Akorn) every 12-18 h to ensure eyes remained open and lubricated throughout the duration of the experiment. For extended eyelid closure experiments, P11 pups were anesthetized and two mattress sutures were placed along the eyelid margin to prevent eye opening as described.^139^ Animals were checked daily to ensure sutured eyes remained closed until euthanasia.

#### Pharmacologic interventions

Mice were anesthetized with xylazine (10 mg/kg, IP) and ketamine (80 mg/kg, IP). Anesthetized mice were injected intravitreally (1–2 μL) with vehicle, balanced salt solution (Alcon, 0065079550), BDNF (3 μg/μL; Peprotech, 450-02), tetrodotoxin (TTX; 3 μM, Abcam, ab120055), or combined TTX (3 μM) and BDNF (3.3 μg/μL). For RGC culture experiments, TTX (1 μM), BDNF (0.05 μg/mL), and cycloheximide (1 μM; EMD Millipore, 239764) or equivalent control DMSO dilutions were prepared in Neurobasal media (Thermofisher Scientific, 21103-049).

#### Cell culture labeling, treatments, and assays

In mitochondrial labeling experiments, purified RGCs are plated and treated with baculoviruses to label mitochondria (BacMam 2.0, Thermo Fisher Scientific, C10601) and 48 h later incubated with pharmacological agents, at stated concentrations and times in text and figure legends. In RNA or protein labeling experiments RGCs were incubated with EU for 2 h or OPP for 15 min (according to the Click-iT Nascent RNA or Protein Synthesis Assay Kit from Thermo Fisher Scientific, C10327 or C10456). After all incubations cells were then fixated (4% PFA PBS), permeabilizated (0.5% Triton PBS), and in EU- or OPP-incubated cells, Click labeling reaction were performed. To identify ribosomes, cells were incubated with antibodies against ribosomal protein S3 (Cell Signaling Technology, D50G7) at 1:100 overnight at 4°C, and secondary Alexa 647 conjugated antibodies at 1:500 for 2hrs at room temperature. To label growth cones, cells were incubated with Alexa Fluor 647 Phalloidin (Thermo Fisher Scientific, A22287) at 1:40 room temp. for 30 min prior to confocal imaging. MTCO1 (1:500 overnight) antibody-stained axons were used as controls in colocalization studies. In oxygen consumption experiments, RGCs were plates as described above, with or without BDNF, at 40k/well in 96 well plates designed for the Seahorse XF96 instrument (Agilent, 101085-004). After culturing for 24hrs, media was exchanged with Assay Media (Agilent, 102365-100) and FluxPak injectable ports were loaded with drugs as recommended by mitochondrial stress test kit (Agilent, 103015-100). TTX was loaded in the empty port A as the first injection, followed by Oligomycin, FCCP, and Rotenone/Antimycin A, respectively. After assay was complete oxygen consumption values were normalized to the number of Dapi positive cells per well.

#### Confocal imaging

B6.Cg-Tg(*Thy1*-CFP/Cox8a)S2Lich/J mice (Jackson labs) were euthanized and perfused with 4% PFA in PBS at P9, P13, P15, and P45. Perfused animals were then enucleated and the eyes and the optic nerves post-fixed in 4% PFA for 1–3 h. Post fixed tissues were whole mounted on slides in Vecta-Shield mounting medium (Vector Labs, #H-1400) and imaged on a Zeiss LSM 710 confocal microscope, all images were taken with the same laser power, gain, and digital offset, at non-saturating levels. Compressed Z-stacks were analyzed by selecting nine random 25 × 50 μm sections and then measuring CFP expression with the ImageJ (National Institutes of Health) particle analyzer tool, after applying a common threshold to images and an automated size threshold on objects larger than 0.08 μm^2^ and smaller than 7 μm^2^ (Figure S1). Thresholding can introduce dimmer mitochondria or out of plane mitochondria as smaller segments, which are removed using size thresholds. Of note, we did not amplify CFP fluorescence with antibodies to avoid increasing background artifacts. Images containing cell bodies were cropped to avoid cell bodies in the analysis. All images of cultured RGCs were collected on a Zeiss LSM 880 confocal system with a 40×/63× objective and using airyscan imaging mode with similar and non-saturating settings, followed by airyscan processing using Zen software. Mander’s colocalization analysis was conducted using Imaris Software 3D-colocalization analysis module (Bitplane), thus allowing us to render more accurate data as co-localization values throughout the entire volume of an image. In addition, mitochondrial encoded MTCO1 antibody staining served as a co-localization positive control for thresholding dsRed labeled mitochondria, as these signals should have perfect biological colocalization. For additional *in vitro* image analysis needs, such as quantifying mitochondrial area, number, number of OPP puncta, and Pearson values between mitochondria and OPP in an axon, Volocity (PerkinElmer) image analysis software was applied using modules identify objects by intensity, intersects with identified object, and/or Pearson correlation values between intersecting objects. Example images of Volocity and Imaris thresholds used to identify mitochondrial area, ribosomes, OPP puncta, MTCO1, and colocalization are presented in Figures S4 and S5.

#### Electron microscopy

Wild type/cousins or littermate control offspring from CD1 crossbred to B6.Cg-Tg(*Thy1*-CFP/Cox8a)S2Lich/J mice perfused under anesthesia with one-half Karnovsky’s fixative; 2.5% glutaraldehyde and 2% paraformaldehyde (PFA) in 0.2M cacodylate buffer. Mice were euthanized and eyes with optic nerves were post fixed in half Karnovsky’s fixative. Tissues were placed in 2% glutaraldehyde overnight and then rinsed in 0.1M phosphate buffer with osmium tetraoxide. Osmicated tissues were rinsed in 0.15M phosphate buffer and dehydrated with graded concentrations of cold ethanol, ranging from 25 to 100%. Dehydrated tissues were rinsed with propylene oxide and embedded in Epon-Araldite with DMP-30 (All reagents were purchased from Electron Microscopy Sciences). Mitochondrial were imaged using a TEM (JEOL JEM1400) and numbers were counted per axon area delineated by well-established ultrastructure morphological features, i.e., myelination, axoplasm, microtubules, and neurofilaments.^93^^,^^94^^,^^95^^,^^96^^,^^97^^,^^98^ Mitochondrial and axon boundaries were manually traced, and the areas were calculated using ImageJ analysis software (example images and equations used in quantification are presented in Figure S2). For immunogold labeling, sectioned were treated as previously described.^140^ Briefly, sections were placed on nickel grids and incubated with a 4% aqueous sodium metaperiodate for 10 min at room temperature, then placed in 4% aqueous sodium metaperiodate for 10 min at room temperature, rinsed with water, placed in 1% aqueous periodic acid for 10 min, rinsed in water again and blocked in PBS (1×) containing 1% BSA, 5% goat serum, 0.1% gelatin, and 0.05% tween 20 for 30 min. Then sections were incubated with ribosomal protein S3 antibody in blocking buffer (at 1:10; Cell Signaling Technology, D50G7) at 4°C overnight, rinsed with PBS-0.05% tween 10 times for 5 min, and incubated with 10 nm immunogold conjugated anti-rabbit antibody (1:50; Abcam, ab27234) for 2 h, followed by a thorough rinsing in water and counterstaining with uranyl acetate and lead citrate prior to TEM imaging. Secondary-only antibody samples served as a control for potential background noise, which was not evident in control images, and areas containing endoplasmic reticulum consistently contained heavy labeling in ribosome antibody stained samples, as expected and suggesting that the antibody was selective for ribosomes in TEM images (Figure S6).

#### Mitochondrial purification

Whole retinas or optic nerves and tracts were quickly dissected from CO2 sacrificed mice, and homogenized using a dounce tissue grinder (Wheaton, 357538) with 20–30 strokes in mitochondrial isolation buffer (provided in the Mitochondria Isolation Kit, Miltenyi Biotec, 130-096-946) with protease inhibitors (Thermofisher Scientific, 78425). The homogenate was then spun at 1000g and the supernatant was removed for subsequent magnet based mitochondrial isolation according to Milteny Biotec’s Mitochondrial Isolation Kit. For Ha tagged mitochondrial captures (MitoTag),^92^ TOM22 antibodies were replaced with an HA magnetically conjugated antibody (Miltenyi Biotec,130-091-122). Isolated mitochondria were washed and re-pelleted three times to insure mitochondrial fractions were pure and intact, for all downstream experiments. In addition, all procedures were performed on ice or at 4°C, to preserve mitochondrial integrity.

#### FACS analyses of mitochondria

Tom22 purified CFP-expressing mitochondria were analyzed with forward and side scatter in a Becton Dickinson FACScan (Becton Dickinson, San Jose, CA). In some experiments, mitochondria were co-labeled with mitotracker CMXROS (Thermofisher Scientific, M7512). To determine if purified mitochondria were intact and viable, FACS sorted mitochondria were equilibrated with the membrane potential–sensitive dye JC-1 (500 nM; 5,5′6,6-tetra-chloro-1,1,3,3-tetraethylbenzimidazol-carbocyanine iodide; Thermofisher Scientific, T3168) for 20 min with or without FCCP (10 μM; carbonylcyanide-P-trifluoromethoxyphenylhydrazone; Sigma Aldrich, C2920). Data were acquired in list mode, and evaluated with WinList software (Verity Software House).

#### Western blots

To further evaluate the structural integrity and purity of isolated mitochondria, mitochondria were analyzed by western blot using the following antibodies. Antibodies against inner and outer mitochondrial membrane integrity proteins include; outer membrane proteins - Porin (VDAC1) and TOM20, inner membrane protein - Ubiquinol Cytochrome *c* Reductase Core Protein I, intermembrane space proteins - Cytochrome *c* and Complex Va, and matrix space protein - Cyclophilin 40, (Abcam-ab110414, ab14734, ab78547, ab110252, ab110325, ab110273, and ab110324). Electron transport chain protein antibodies include; Complex I subunit (NDUFB8), Complex II-30kDa (SH3B), Complex III-Core Protein 2 (UQCRC2), Complex IV subunit I (MTCO1), and Complex V alpha subunit (ATP5A) (Abcam-ab110411; ab110242, ab14714, ab14745, ab14705, and ab14748). Ribosomes were also detected in purified mitochondrial fractions using ribosomal protein S3 antibody (Cell Signaling Technology, D50G7). Cytoplasmic and nuclear antibodies included GAPDH and β-Actin (Cell Signaling Technology, 2118S and 8457). Protein samples were loaded equally for all Westerns based on BCA protein content detection. In proteinase K-treated comparison experiments rather than rely on BCA, which would detect the spiked-in proteinase K itself, individual mitochondrial preps were instead split into equal volumes and treated equally prior to loading equivalent volumes in blots. Ponceau S staining confirmed relatively similar protein loading per lane.

#### RNA detection

To detect mitochondrial or nuclear-encoded mRNA transcripts, RNA was extracted from isolated RGCs, whole retinas, or mitochondria from retina or optic nerve and tract using the RNeasy Plus Micro Kit (Qiagen, 74034). RNA isolates were then processed for RT^2^ Profiler PCR Arrays (Mouse Mitochondrial and Mitochondria Energy Metabolism 330231, PAMM-087ZE and PAMM-008ZE). The resulting data was passed through IPA pathway analysis software (Qiagen), for upstream regulator identification and visual representation in heat maps (Data S1). For mitochondrial RNA release assays, isolated mitochondria from P15 retinas were resuspended in mitochondrial suspension buffer (provided in Mitochondria Isolation Kit) and incubated with proteinase K (Thermofisher Scientific, 25530049) at 5 μg/mL and/or 200 ng of total RNA isolated from P15 retinas for 5 min. Mitochondria were then pelleted, and supernatant and mitochondrial pellets were separately processed for RNA purification, subsequent RT steps (Biorad, 1708891), and qPCRs (Thermofisher Scientific, 4369016). All qPCR data were acquired on QuantStudio 7 Flex Real-Time PCR System (Applied Biosystems, Thermofisher Scientific). Taqman gene expression primers include (Thermofisher Scientific); *Mtfp1*-Mm00466042_m1; *Snph*-Mm01243855_m1; *Cox6b2*-Mm01333764_g1; *Pink1*-Mm00550827_m1; *Pum1*-Mm01180596_m1, *Pum2*-Mm00472901_m1; *Rhot2*-Mm00524478_m1; *Rhot1*-Mm01304158_m1; *Opa1*-Mm01349713_m1; *Opa3*-Mm 01313640_m1; *Fis1*- Mm00481580_m1; *Mfn1*-Mm00612599_m1; *Mfn2*-Mm00500120_m1; *Mtfr1*-Mm01289320_m1; *Dnm1l*-Mm01342903_m1; *Ppargc1a*-Mm01208835_m1; *Cox8a-*Mm02342396_g1; *Rnr2*-Mm04260181_s1; *Nd4*-Mm04225294_s1; *Actb*-Mm04394036_g1; *Gapdh*- Mm99999915_g1.

#### Proteomics

To detect mitochondrial associated proteins, mitochondrial purification was performed on 3 replicate optic nerve and 3 replicate retina mitochondrial isolations, from young mice (ages P15-20, 3 retinas and 18–24 optic nerves per replicate prep). Resulting mitochondrial pellets were solubilized with 5% rapigest in TNE buffer and then boiled for 5 min, followed by reduction in 1 mM Tris(2-carboxyethyl)phosphine hydrochloride at 37°C for 30min. Then samples were alkylated in .5 mM 2-iodoacetamide at 37°C for 30min, followed by trypsin digestions at 1:50 (enzyme:protein) overnight at 37°C and the addition of 250 mM HCl at 37°C for 1h. Samples were then centrifuged and peptides were extracted from the supernatant and desalted using Aspire RP30 desalting columns (Thermo Scientific).^141^ Trypsin-digested peptides were analyzed by LC-MS/MS^142^ on the TripleTOF 5600 hybrid mass spectrometer (ABSCIEX). MS/MS data were acquired in a data-dependent manner in which the MS1 data was acquired for 250 ms at m/z of 400–1250 Da and the MS/MS data was acquired from m/z of 50 to 2,000 Da. For Independent data acquisition (IDA) parameters MS1-TOF 250 ms, followed by 50 MS2 events of 25 ms each. The IDA criteria; over 200 counts threshold, charge state of plus 2–4 with 4 s exclusion window. Finally, the collected data were analyzed and normalized^143^ using MASCOT (Matrix Sciences) and Protein Pilot 4.0 (ABSCIEX) for peptide identifications normalized based on spectral abundance factors. Peptides were considered significantly present when detected with >95% confidence, 1 or more times within a prep. Description and source data of the filtering methods applied as well as raw data, are present in Data S2 and S3.

#### RNA-seq and analysis

RNA isolated from purified mitochondria, were submitted to the Stanford Genomic Sequencing Service Center for the Total RNA Prep, including Ribo-zero depletion of rRNA is followed by strand-specific library preparation using the TruSeq Stranded Total RNA Library Preparation kit. (Illumina). Differential expression analysis between samples was performed in R version 3.4 using the DESeq2 package.^144^ Low expression genetic features were removed after alignment with CPM value ≤ 1 in less than 2 samples. Genes with a false discovery rate (FDR) adjusted *p*-value <0.05 and a log2 fold change >1 or < −1 were considered significantly differentially expressed. Heatmaps of differentially expressed genes were created using the online tool Morpheus. Volcano, dot and bar plots were generated with custom scripts using ggplot2 in R. To identify enriched pathways among the differentially expressed genes, GO term enrichment analysis was conducted using g:Profiler. This analysis included biological process and cellular component categories. Enriched GO terms with an adjusted *p*-value <0.05 were considered significant. Mitochondrial-related genes were identified using the HGI Quick Search tool. This tool searches for terms, synonyms, and definitions from various vocabularies. By searching for the keyword ‘mitochondria,’ we identified 2,012 genes associated with mitochondrial functions. These genes were then cross-referenced with our differentially expressed gene list to identify mitochondrial-related transcripts. Statistical analyses were performed using R and Bioconductor packages. The Mann-Whitney U test was used to compare the expression ranks of mitochondrial and non-mitochondrial genes, with a significance threshold set at *p* < 0.05.

### Quantification and statistical analysis

#### Graphing and statistics

Data presentation and statistical analysis was done in Prism (Graphpad). To compare quantitative variables, Student’s t-tests or ANOVA with post-hoc t-tests were done with a *p*-value <0.05 indicating statistical significance. Additional detail on specific statistical tests applied along with graphed averages derived from number of animals, cells, or samples tested are provided in figure legends for each figure. Additional details on software and equations applied in data capture and quantification are indicated in main figure or supplemental figure legends. All quantified data presented in bar charts or plots along with statistical testing and representations, was created in Prism (GraphPad). Infographics were created with either modified Motifolio and/or BioRender curated images.

## Supplemental information

Document S1. Figures S1–S8

Data S1. Raw data used for IPA based heatmap renderings and upstream analysis for Figures 5B, 5C, and 5E

Data S2. Filtering procedures applied to proteomics data, as described in Figure 9

Data S3. Raw data from the visual system mitochondrial proteome in Figure 9

Data S4. Source Data for Figure 10B–10H
