# Supplementary material for: Light-evoked activity and BDNF regulate mitochondrial dynamics and mitochondrial localized translation in CNS axons
Source: iScience. 2025 Sep 15;28(10):113563. doi: 10.1016/j.isci.2025.113563 (PMC12547173; doi:10.1016/j.isci.2025.113563)
Supplement: Document S1. Figures S1–S8 [file mmc1.pdf]

iScience, Volume *28*

Supplemental information

Light-evoked activity and BDNF regulate mitochondrial dynamics and mitochondrial localized translation in CNS axons

Alexander Kreymerman, Jessica E. Weinstein, Nirmal Vadgama, Sahil H. Shah, Michael M. Nahmou, Kinsley C. Belle, Marco H. Ji, Xin Xia, Anne Faust, Yolandi Van Der Merwe, David N. Buickians, In-Jae Cho, Star K. Huynh, Sonya Verma, Kristina Russano, Xiao-Lu Jin, Ioannis Karakikes, Michael B. Steketee, and Jeffrey L. Goldberg

a

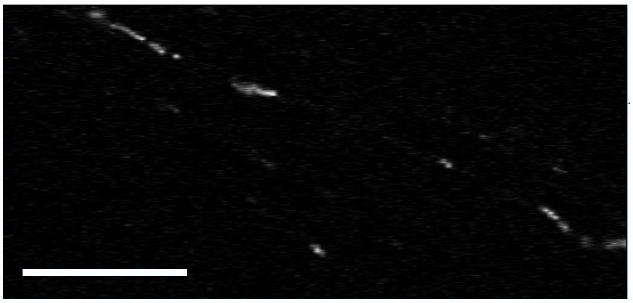

b

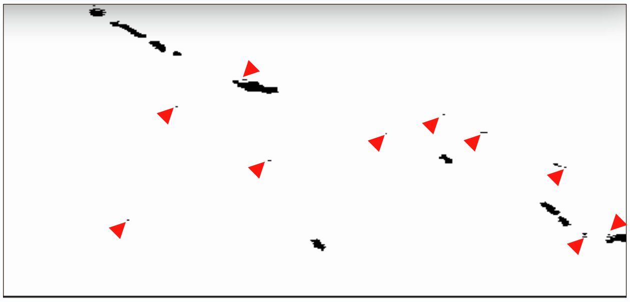

c

Imaging	Average Mito. Size	Average # of Mito.	Area Occupied by Mito. (%)
Confocal	Mito. Area per image Z /#Mito per Z	# Mito. per Z /Total Image area per Z	(Mito. Area per image Z/ Total Image Area per Z)*100

Supplementary Figure 1. Analysis approach for quantifying CFP+ mitochondria in vivo. (**a**) Example cropped image of CFP+ mitochondria in a single plane of a confocal imaged retinal axon. (**b**) Threshold mask applied in ImageJ prior to using the analyze particles function to identify mean area of CFP+ mitochondria in a given confocal slice (5µm scale bar). Red arrows indicate threshold fragments smaller than .08µm^2 that are removed by the analyze particles function. (**c**) Tabled equations used to identify graphed values in confocal images of mitochondria.

a

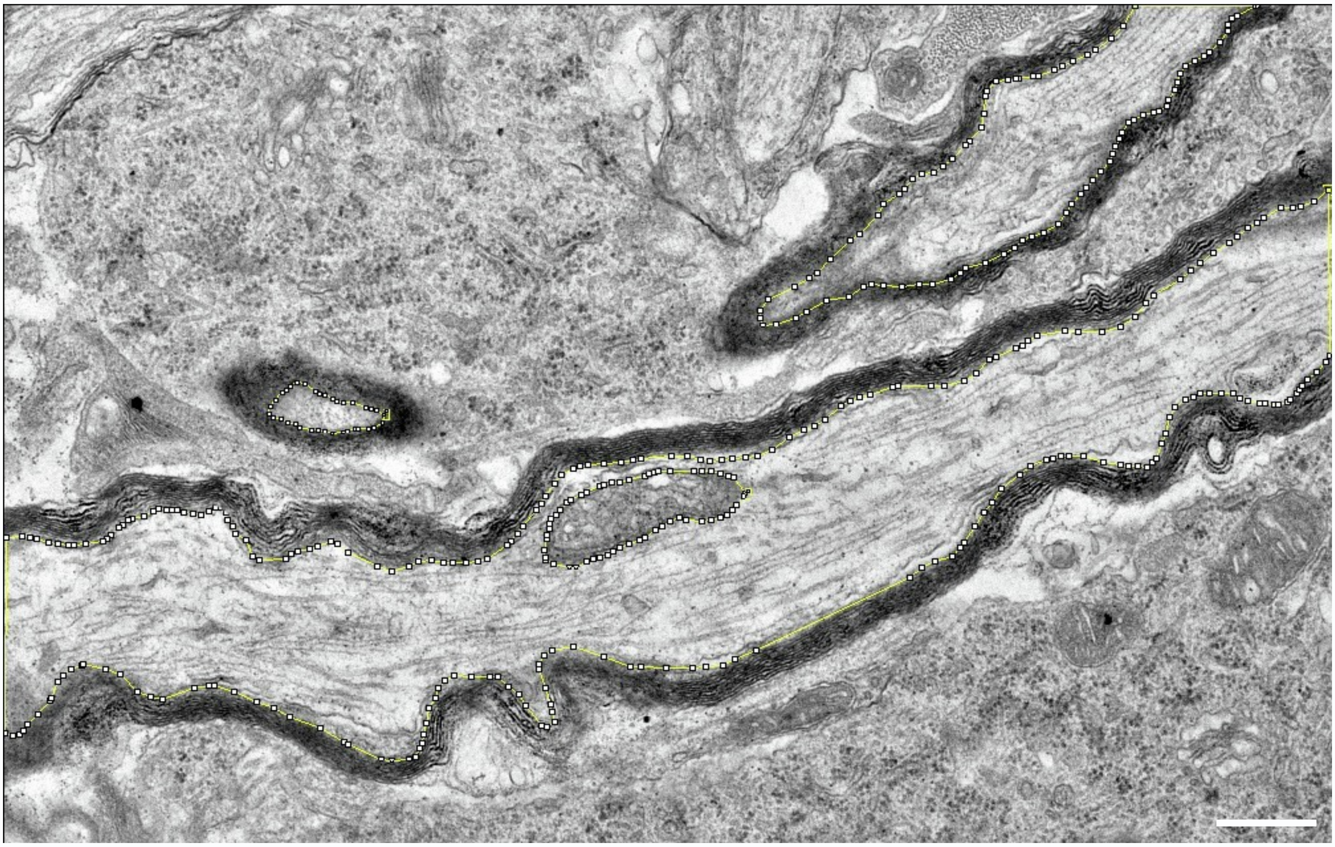

b

Imaging	Average Mito. Size	Average # of Mito.	Area Occupied by Mito. (%)
TEM	Mito. Area per image /#Mito per image	(# Mito./Axon Area)/(Total Image area)	((Mito. Area per image/ Total Axon Area)/Total Image area)*100

Supplementary Figure 2. Analysis approach for quantifying mitochondria in TEM images. (**a**) Example transmission electron microscope image of an optic nerve section. Tracing showing an axon, identified by myelination and neurofilament structures, as well as and an intra-axonal mitochondria (500nm scale bar). (**b**) Tabled equations used to identify graphed values in TEM images of mitochondria.

a

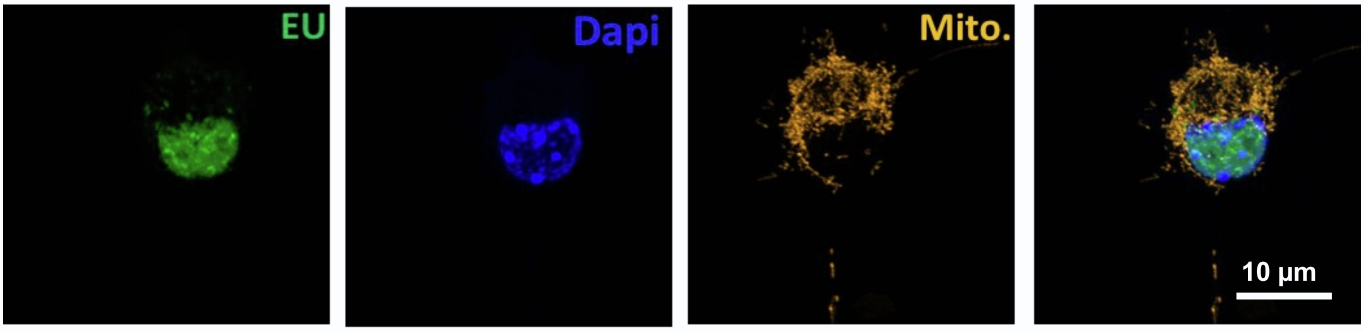

Supplementary Figure 3. EU labeling in RGCs presents as an intense perinuclear signal in somas. (**a**) Confocal images of cultured P4 RGCs treated with BacMam virus, labeling mitochondria with DsRed, and pulsed with EU for 1hr before fixation and staining for newly synthesized RNA. EU RNA staining was strongly detected in RGC nuclei and cell bodies, but not in axons.

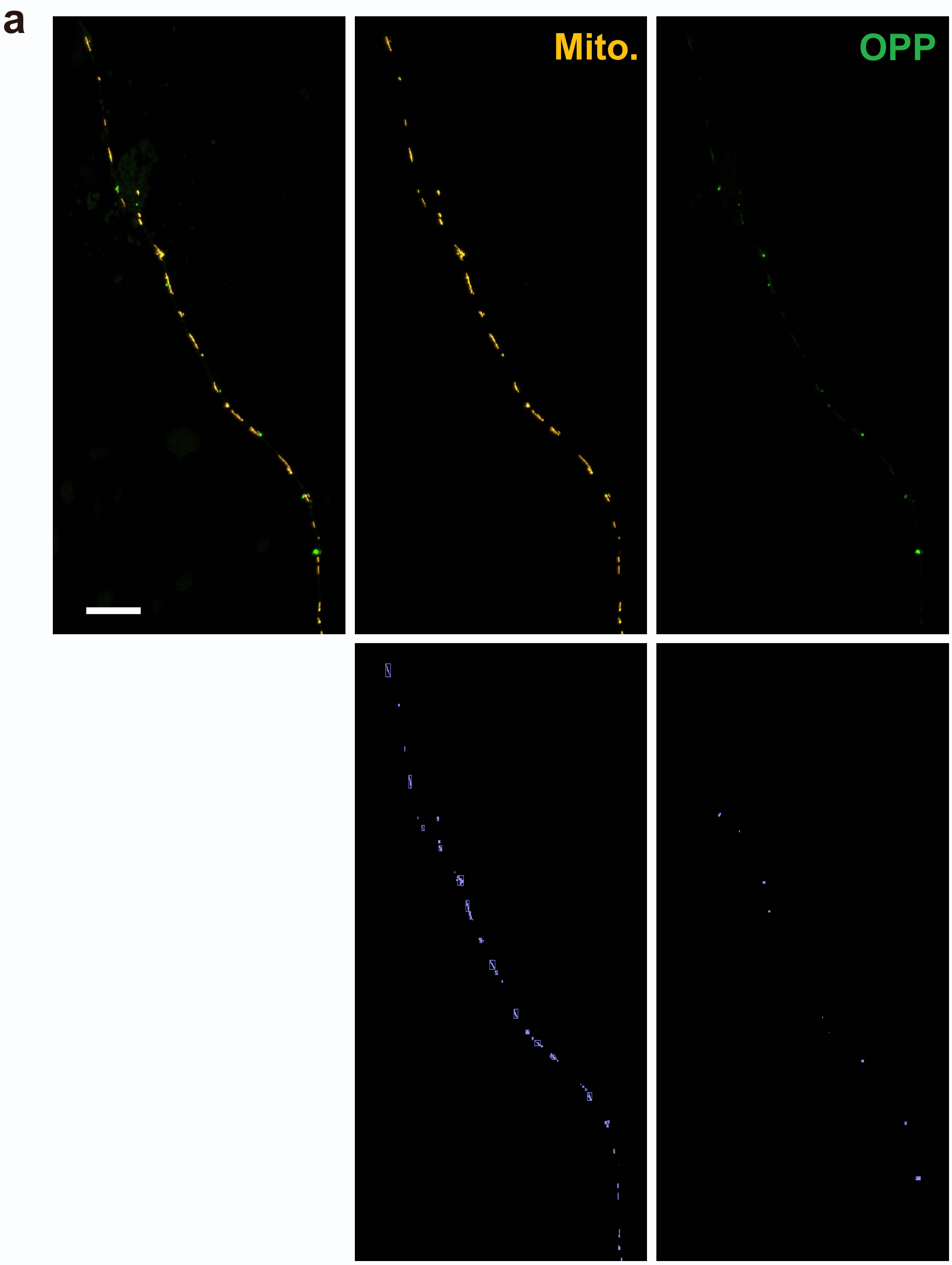

Supplementary Figure 4. Approach for identifying and quantifying mitochondria and OPP signals. (**a**) An example maximum intensity projection of an axon in a cultured RGC, containing DsRed viral labeling of mitochondria (Mito.) and OPP labeling of protein synthesis (Alexa488, 10um scale bar). Top images are of native fluorescent imaging results and corresponding bottom images are example thresholding set for masking and identifying Mito. and OPP signals in Volocity's identify objects by intensity module. Masks were used to calculate mean mitochondrial area in axons along with Pearson correlation values for Mito-OPP association, using Volocity's intercepts module.

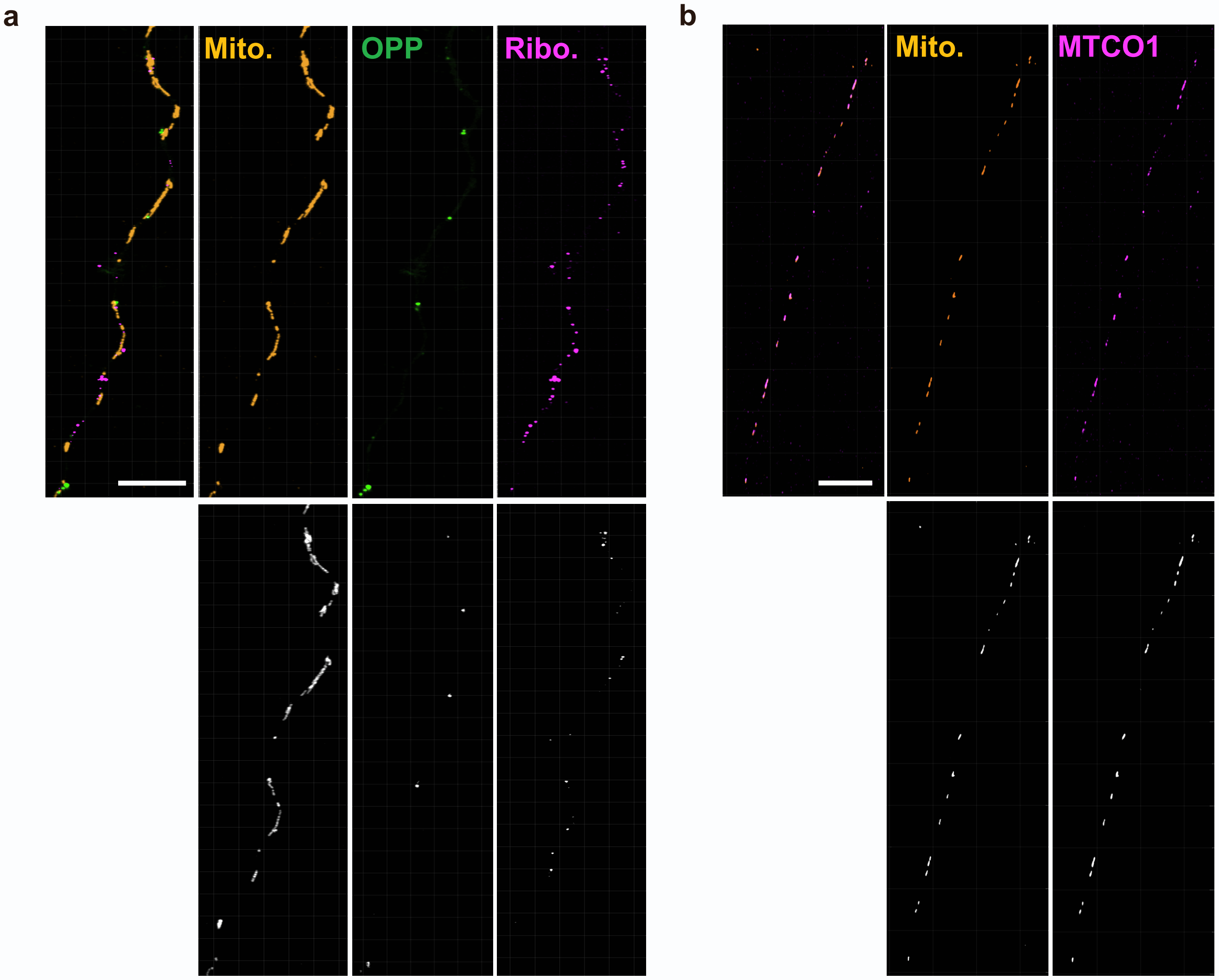

c

	Mito.-OPP	Mito.-Ribo	Mito.-MTCO1
**Mean Mander's Coefficient**	0.3586	0.2868	0.9625
**Standard Error Mean**	0.03162	0.03185	0.008889

Supplementary Figure 5. Analysis approach for OPP, Ribosomal, and mitochondria colocalization. (**a**) An example maximum intensity projection of an axon in a cultured RGC, containing DsRed viral labeling of mitochondria (Mito.), OPP labeling of protein synthesis (Alexa488), and ribosomal protein S3 (Ribo.) antibody staining (Alexa647, 10um scale bar). Top images are of native fluorescent imaging results and corresponding bottom images are example thresholds used to mask Mito., OPP, or Ribo., by Imaris's colocalization module. Masks in the OPP and Ribo channels represent colocalization sites with mitochondria and form the basis for Mander's Coefficient values identified by Imaris. (**b**) A representative image of Mito. labeling and MTCO1 antibody staining in RGC axons. MTCO1 staining serves as a control for colocalization parameters set in Imaris's colocalization module and shows perfect matching with Mito., as represented in the corresponding mask of the colocalized portion in the MTCO1 channel (10um scale bar). (**c**) Tabled mean Mander's coefficient values along with corresponding SEM values as identified in figure 6H, with added values for Mito.-MTCO1 colocalization.

a

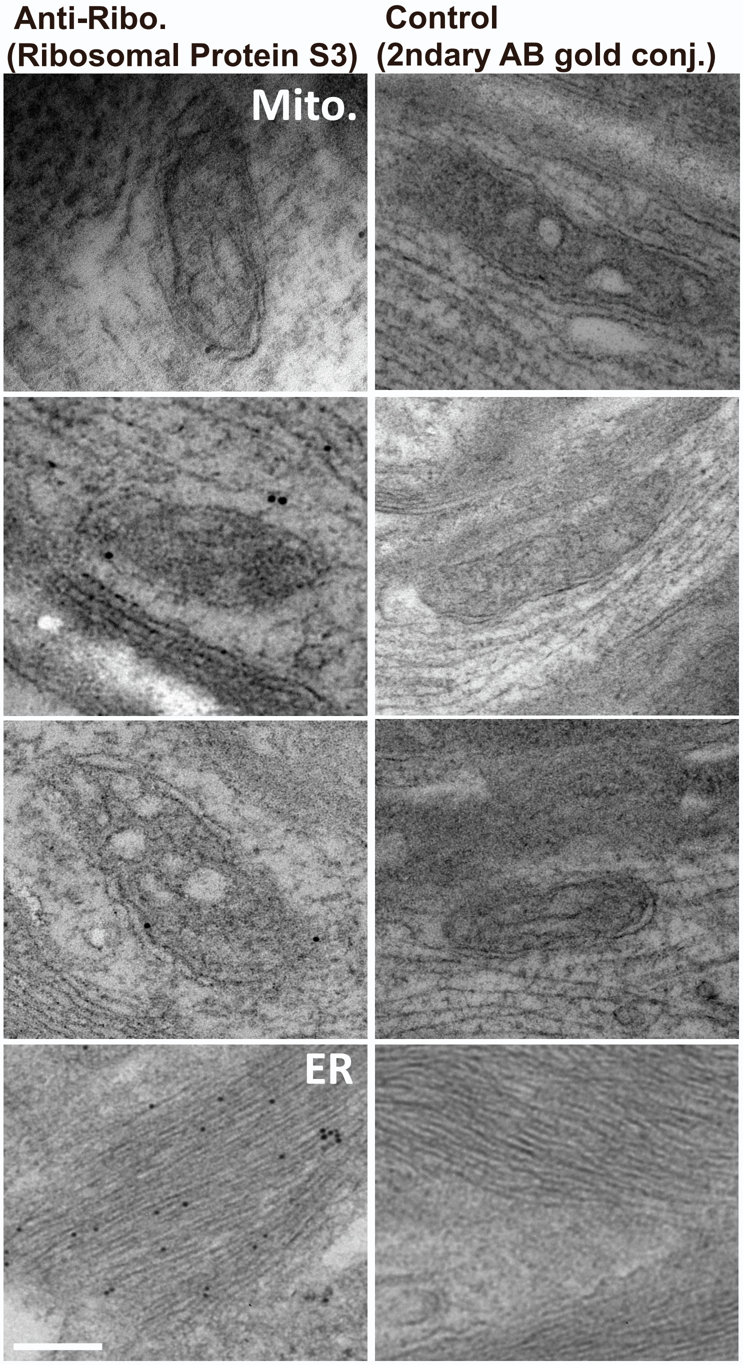

b

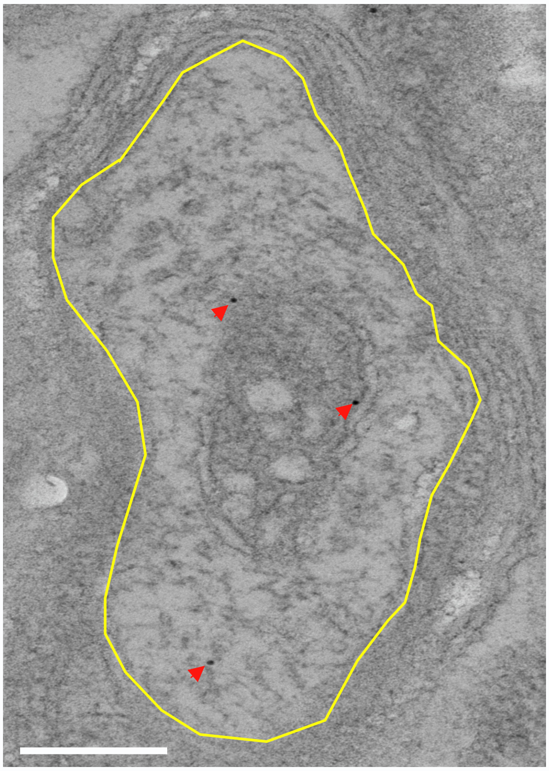

Area (um²)	Particles Associated with Mito.	Total Particles
.571	2	3

c

% of Particles Associated to Mito. Normalized to Axon Area =((particles on mito/ total particles)*axon area)*100%

% Particles Assoc. with Mito.	STDEV
37.7% (n=6 sections)	11.9

Supplementary Figure 6. Analysis approach for OPP and mitochondrial colocalization in TEM. (**a**) TEM images of ribosomal protein S3 (Ribo.) labeling in P15 optic nerve sections, identified using a secondary antibody for the Ribo. antibody with a conjugated 10nm gold particle (200nm scale bar). Left column contains primary and secondary antibody labeling and right column contains control images of secondary only stained samples. Top 3 panels show zoomed images of mitochondria and bottom panels are of endoplasmic reticulum (ER). Left column images show gold particles bound to mitochondrial outer membranes as well as throughout the ER (arrows point to particles), a known site of ribosomal localization. No identifiable gold particles where found in the control column. (**b**) A representative image of a Ribo. stained optic nerve axon, identified by myelination and neurofilament structures. Area is traced (yellow lines) and ribosomes counted (red arrows) in ImageJ (200nm scale bar), along with corresponding values underneath the image. (**c**) Normalized values for the % of particles associated with Mito. were quantified by normalizing against the total number of particle per axonal area. Tabled values represent quantifications from n=6 stained optic nerve axonal sections and 18 mitochondria.

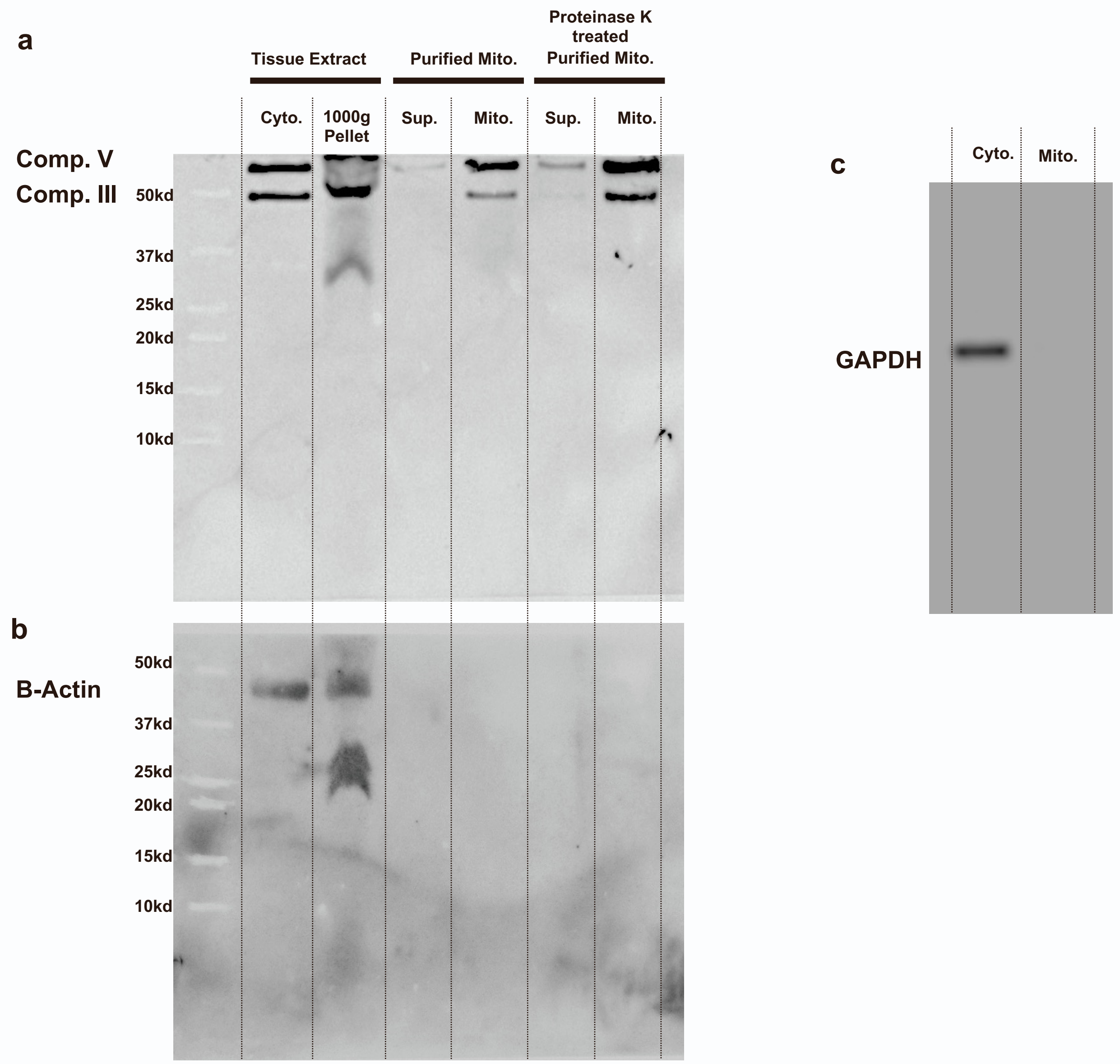

. Analysis approach for OPP, Ribosomal, and mitochondria colocalization.

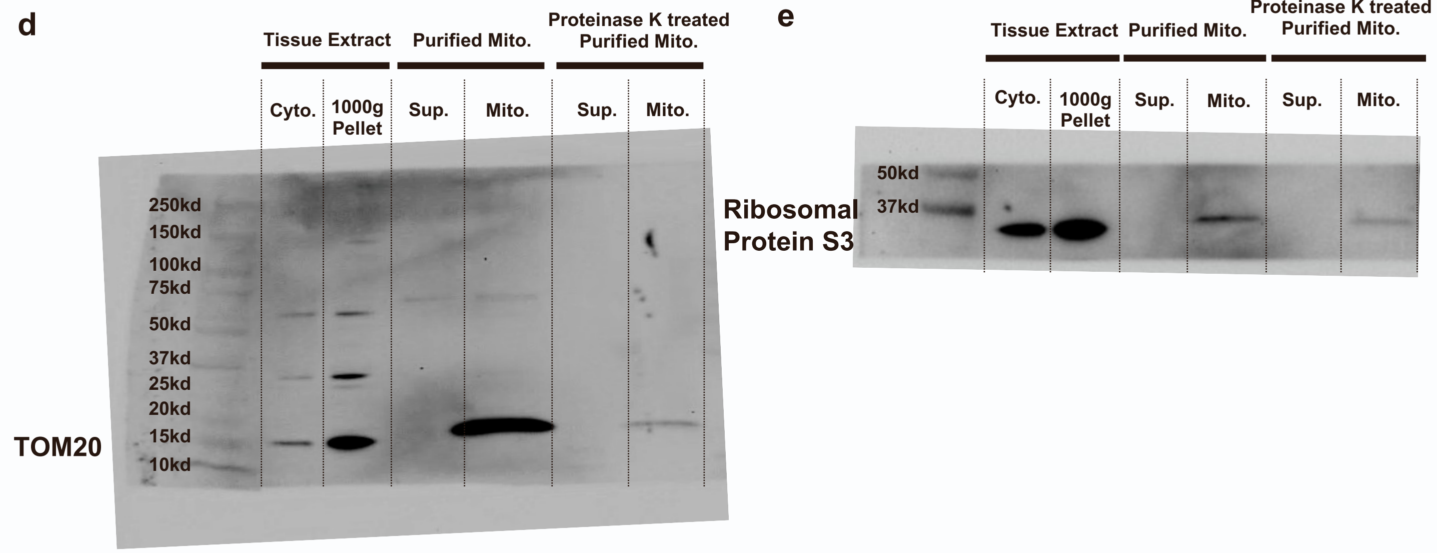

Supplementary Figure 7. Images of full western blots from Figure 7, panel e and b.

Images of the supplemental blots or entire membranes from western blots of mitochondrial enrichment, cropped images shown in Fig. 7e and Fig. 8b. Samples loaded as presented in the images. (**a**) and (**b**) are uncut versions of blots from Fig. 7e and Fig. 8b. The same membrane was blotted sequentially first for Complex III-Core Protein 2 and Complex V alpha subunit (mouse antibodies) and then with b-actin (rabbit antibody). Labels are placed at the corresponding molecular weights of the expected bands. Molecular weights are marked by Precision Plus Protein Kaleidoscope Prestained Protein Standards. (**c**) Supplemental blot showing that mitochondrial isolates are purified from common cytoplasmic contaminating protein GAPDH. (**d**) Uncut blot from Fig. 8b, blotted for TOM20. Strong banding is present at the expected molecular of 16kd, as specified by the manufacture (Abcam, ab78547), along with additional unknown upper bands. (**e**) Uncropped blot for ribosomal protein s3, with distinct banding at ~30kd. The membrane was cut at ~ 60kd and just above 20kd for additional probing procedures.

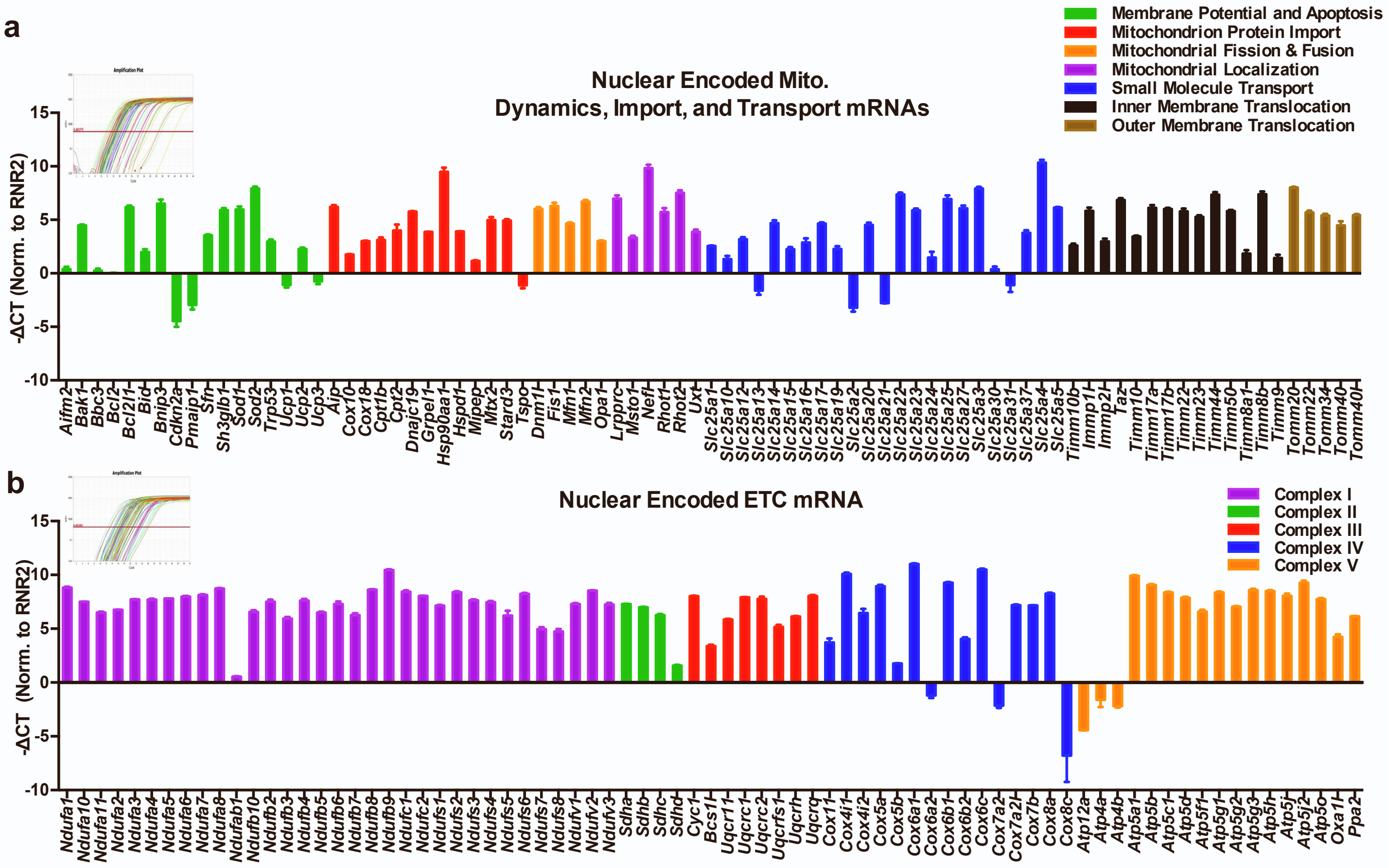

Supplementary Figure 8. Data supporting that nuclear mRNAs are detected and present in isolated mitochondria. Isolated mitochondria bind nuclear-encoded RNAs associated with mitochondrial dynamics and energetics. (**a**) qRT-PCR array of mitochondrial dynamics and (**b**) energetics genes from purified mitochondria, normalized to RNR2, a mitochondria-encoded ribosomal RNA (Error bars represent SEM; N=3 retina mitochondria purifications). Subpanel in the upper left corner shows that all CT values were pulled from amplifications < 30 cycles. The data is presented as the inverse of the delta CT value, indicating more or less abundance of a particular nuclear encoded gene relative to RNR2. Genes are clustered by annotated functional roles in mitochondria.
